# Deciphering Complex Interactions Between LTR Retrotransposons and Three *Papaver* Species Using LTR_Stream

**DOI:** 10.1093/gpbjnl/qzaf061

**Published:** 2025-07-08

**Authors:** Tun Xu, Stephen J Bush, Yizhuo Che, Huanhuan Zhao, Tingjie Wang, Peng Jia, Songbo Wang, Peisen Sun, Pengyu Zhang, Shenghan Gao, Yu Xu, Chengyao Wang, Ningxin Dang, Yong E Zhang, Xiaofei Yang, Kai Ye

**Affiliations:** School of Automation Science and Engineering, Faculty of Electronic and Information Engineering, Xi’an Jiaotong University, Xi’an 710049, China; MOE Key Lab for Intelligent Networks & Networks Security, Faculty of Electronic and Information Engineering, Xi’an Jiaotong University, Xi’an 710049, China; School of Automation Science and Engineering, Faculty of Electronic and Information Engineering, Xi’an Jiaotong University, Xi’an 710049, China; School of Automation Science and Engineering, Faculty of Electronic and Information Engineering, Xi’an Jiaotong University, Xi’an 710049, China; MOE Key Lab for Intelligent Networks & Networks Security, Faculty of Electronic and Information Engineering, Xi’an Jiaotong University, Xi’an 710049, China; Center for Mathematical Medical, The First Affiliated Hospital of Xi’an Jiaotong University, Xi’an 710061, China; Center for Mathematical Medical, The First Affiliated Hospital of Xi’an Jiaotong University, Xi’an 710061, China; Department of Gynecology and Obstetrics, Center for Mathematical Medical, The First Affiliated Hospital of Xi’an Jiaotong University, Xi’an 710061, China; School of Automation Science and Engineering, Faculty of Electronic and Information Engineering, Xi’an Jiaotong University, Xi’an 710049, China; MOE Key Lab for Intelligent Networks & Networks Security, Faculty of Electronic and Information Engineering, Xi’an Jiaotong University, Xi’an 710049, China; School of Automation Science and Engineering, Faculty of Electronic and Information Engineering, Xi’an Jiaotong University, Xi’an 710049, China; MOE Key Lab for Intelligent Networks & Networks Security, Faculty of Electronic and Information Engineering, Xi’an Jiaotong University, Xi’an 710049, China; School of Automation Science and Engineering, Faculty of Electronic and Information Engineering, Xi’an Jiaotong University, Xi’an 710049, China; MOE Key Lab for Intelligent Networks & Networks Security, Faculty of Electronic and Information Engineering, Xi’an Jiaotong University, Xi’an 710049, China; School of Automation Science and Engineering, Faculty of Electronic and Information Engineering, Xi’an Jiaotong University, Xi’an 710049, China; MOE Key Lab for Intelligent Networks & Networks Security, Faculty of Electronic and Information Engineering, Xi’an Jiaotong University, Xi’an 710049, China; School of Life Science and Technology, Xi’an Jiaotong University, Xi’an 710049, China; Genome Institute, The First Affiliated Hospital of Xi’an Jiaotong University, Xi’an 710061, China; Department of Endocrinology, The First Affiliated Hospital of Xi’an Jiaotong University, Xi’an 710061, China; Genome Institute, The First Affiliated Hospital of Xi’an Jiaotong University, Xi’an 710061, China; Key Laboratory of Zoological Systematics and Evolution, Institute of Zoology, Chinese Academy of Sciences, Beijing 100101, China; MOE Key Lab for Intelligent Networks & Networks Security, Faculty of Electronic and Information Engineering, Xi’an Jiaotong University, Xi’an 710049, China; Genome Institute, The First Affiliated Hospital of Xi’an Jiaotong University, Xi’an 710061, China; School of Computer Science and Technology, Faculty of Electronic and Information Engineering, Xi’an Jiaotong University, Xi’an 710049, China; School of Automation Science and Engineering, Faculty of Electronic and Information Engineering, Xi’an Jiaotong University, Xi’an 710049, China; MOE Key Lab for Intelligent Networks & Networks Security, Faculty of Electronic and Information Engineering, Xi’an Jiaotong University, Xi’an 710049, China; Center for Mathematical Medical, The First Affiliated Hospital of Xi’an Jiaotong University, Xi’an 710061, China; School of Life Science and Technology, Xi’an Jiaotong University, Xi’an 710049, China; Genome Institute, The First Affiliated Hospital of Xi’an Jiaotong University, Xi’an 710061, China; Faculty of Science, Leiden University, 2311 EZ, Leiden, The Netherlands

**Keywords:** LTR-RT clustering, *Papaver*, Horizontal transfer, LTR-RT burst, TAD-like structure

## Abstract

Long terminal repeat retrotransposons (LTR-RTs), a major type of class I transposable elements, are the most abundant repeat element in plants. The study of the interactions between LTR-RTs and the host genome relies on high-resolution characterization of LTR-RTs. However, for non-model species, this remains a challenge. To address this, we developed LTR_Stream for sublineage clustering of LTR-RTs in specific or closely related species, providing higher precision than current database-based lineage-level clustering. Using LTR_Stream, we analyzed *Retand* LTR-RTs in three *Papaver* species. Our findings show that high-resolution clustering reveals complex interactions between LTR-RTs and the host genome. For instance, we found that autonomous *Retand* elements could spread among the ancestors of different subgenomes, like retroviral pandemics, enriching genetic diversity. Additionally, we identified that specific truncated fragments containing transcription factor motifs such as TCP and bZIP may contribute to the generation of novel topologically associating domain-like (TAD-like) boundaries. Notably, our pre-allopolyploidization and post-allopolyploidization comparisons show that these effects diminished after allopolyploidization, suggesting that allopolyploidization may be one of the mechanisms by which *Papaver* species cope with LTR-RTs. We demonstrated the potential application of LTR_Stream and provided a reference case for studying the interactions between LTR-RTs and the host genome in non-model plant species.

## Graphical abstract

**Figure:**
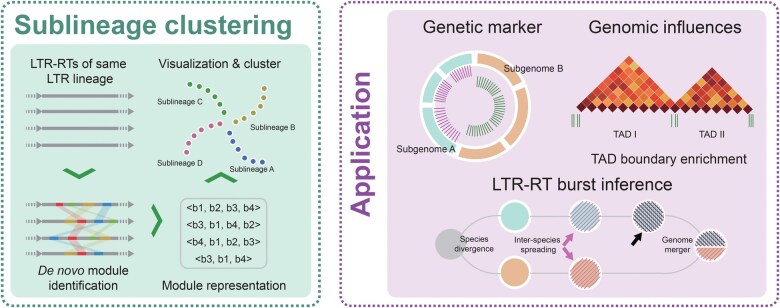

## Introduction

Transposable elements (TEs), also known as transposons, are mobile DNA elements that can cut or copy and insert themselves into different locations within a genome. In humans and other model organisms, extensive research has led to well-established TE classification systems [1]. For instance, human Alu elements can be categorized into 213 subfamilies [2]. This refined classification forms the foundation for studying their complex evolutionary history [2] and interactions with host genomes [3–6]. In plants, long terminal repeat retrotransposons (LTR-RTs) are the most common type of TEs [7], contributing significantly to the diversity and size variations observed between plant genomes [8–10]. However, unlike in humans and other model organisms, the fine-scale classification of transposons in non-model plants is still challenging, hindering further investigation into the impacts of specific TE types on host genomes. Especially in recent times, with advancements in sequencing and assembly technologies [11], researchers are generating an increasing number of complete plant genomes [8,12–16], leading to a surging demand for the precise classification of LTR-RTs.

For example, our previously published genomes of three *Papaver* species, *Papaver rhoeas* (*P*. *rh*),* Papaver somniferum* (*P*. *so*), and *Papaver setigerum* (*P*. *se*) [13], recently underwent varying degrees of allopolyploidization, and contain one, two, and four subgenomes, respectively. These subgenomes have undergone complex processes of fission and fusion. However, as LTR-RTs constitute the largest proportion of sequences in these genomes [13], their intricate interactions with host genomes under such a complex evolution scenario, remain unclear. For non-model organisms, current methods for classifying transposons, such as DeepTE [17] and TEsorter [18], typically categorize LTR-RTs into broad groups based on their family or lineage (lineage level), which lack the resolution to uncover subgenome-specific LTR-RTs. To address this, we developed LTR_Stream, a tool for fine-scale clustering of LTR-RTs in specific or closely related species. LTR_Stream represents LTR-RTs by identifying conserved sequence fragments (modules) within homology-based networks and clusters them using a graph-based learning approach. While constructing phylogenetic trees based on conserved protein domains within LTR-RTs is a commonly used method [19], non-autonomous LTR-RTs often lack certain protein domains [20], making it challenging to unify them into a single tree for clustering. LTR_Stream overcomes this limitation. Additionally, the modules identified by LTR_Stream can be used to identify and differentiate fragmented LTR-RTs.

Using LTR_Stream, we subdivided the most abundant *Retand* LTR-RT lineage in three *Papaver* genomes into 20 sublineages. At this resolution, we systematically compared the dynamic changes of LTR-RTs across different subgenomes. For instance, we identified molecular genetic markers that provide evidence of *Retand* element spreading between species in a retrovirus-like manner. By leveraging modules identified by LTR_Stream, we detected fragmented LTR-RTs in the whole genome, uncovering their activity diversity across subgenomes and their genomic impacts, such as influencing topologically associating domain-like (TAD-like) structures. Our analysis of *Papaver* species using LTR_Stream provides a reference case for studying the interactions between LTR-RTs and host genomes in non-model organisms. We believe LTR_Stream has significant potential to uncover previously unknown evolutionary mechanisms of LTR-RTs in other non-model species.

## Results

### Overview of LTR_Stream

**Figure 1:**
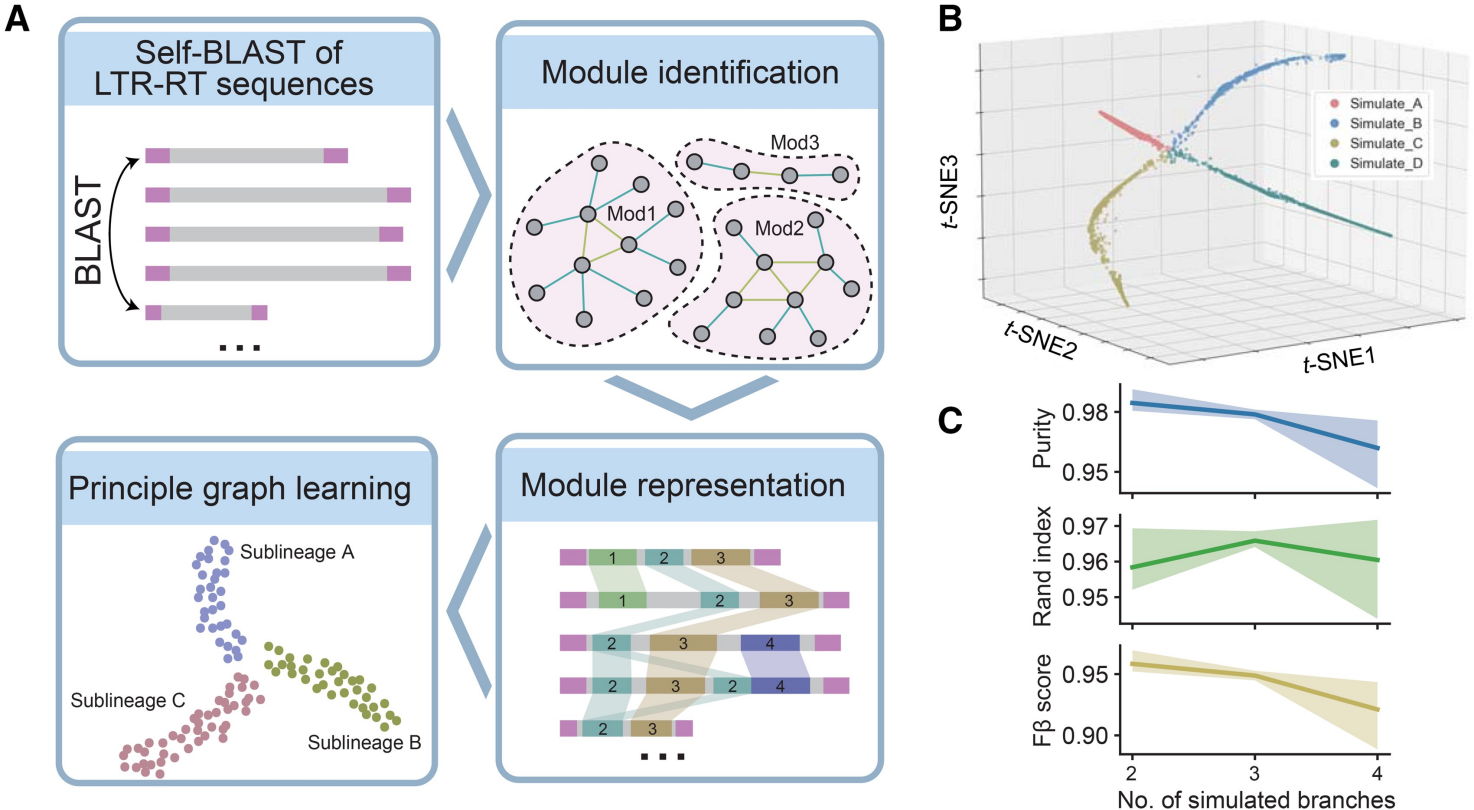
Workflow of LTR_Stream and its performance on simulated datasets **A**. Flow chart showing the four main procedures of LTR_Stream. **B**. 3D dot plot showing the reconstructed trajectories of LTR-RTs on one simulated dataset with four simulated paths. Each dot represents one module sequence that is extracted from one or several LTR-RTs. Colors of dots indicate original simulated path IDs. **C**. Line plots showing clustering performance on nine simulated datasets. LTR-RT, long terminal repeat retrotransposon; mod, module; *t*-SNE, *t*-distributed stochastic neighbor embedding; 3D, three-dimensional; ID, identifier.

LTR_Stream is designed to cluster LTR-RTs at the sublineage level, providing a more nuanced view compared to traditional methods (**Figure 1**A). It takes as input the nucleotide sequences of intact LTR-RTs (those containing two identical or very similar LTRs) belonging to the same LTR lineage. While it can theoretically handle a mix of LTR-RTs from different LTR lineages, using elements from a single LTR lineage is recommended for optimal results. LTR_Stream initially performs self-BLAST with these LTR-RTs, using the results to construct a homology graph. By detecting the connectivity of the graph, LTR_Stream segments each LTR-RT into distinct modules (Figure S1; see Materials and methods), which represent potentially functional or structural conserved regions shared among part of LTR-RTs. LTR_Stream then simplifies the representation of an LTR-RT as a sequential order of modules (module sequence) that reflects the order and composition of LTR-RTs. To cluster LTR-RTs, LTR_Stream calculates a distance matrix by estimating pairwise Levenshtein distances [21] between module sequences and then reduces it to a three-dimensional (3D) space. Finally, a self-designed layer-by-layer clustering algorithm identifies sublineages of LTR-RTs within this space (see Materials and methods).

### Evaluation of LTR_Stream on simulated and real-world datasets

To assess LTR_Stream’s ability to accurately cluster LTR-RTs at the sublineage level, we first tested it on nine sets of simulated data (Table S1). We began by random selection of three real LTR-RT sequences (*Ale*, *CRM*, and *Tork*) from the *Gossypium herbaceum* genome [14]. Each of these sequences was considered the starting point (ancestor) for each simulated dataset. Next, we created simulated independent evolutionary paths for these LTR-RTs, mimicking the four types of changes [bursts, inserions/delettions (indels), structural variations, and elimination] (Figure S2). LTR_Stream successfully distinguished the different simulated evolutionary paths (Figure 1B, Figures S3–S5). Various metrics commonly used to evaluate clustering performance (purity, Rand index, and Fβ score) confirmed LTR_Stream’s effectiveness in correctly clustering simulated LTR-RTs (Figure 1C).

**Figure 2:**
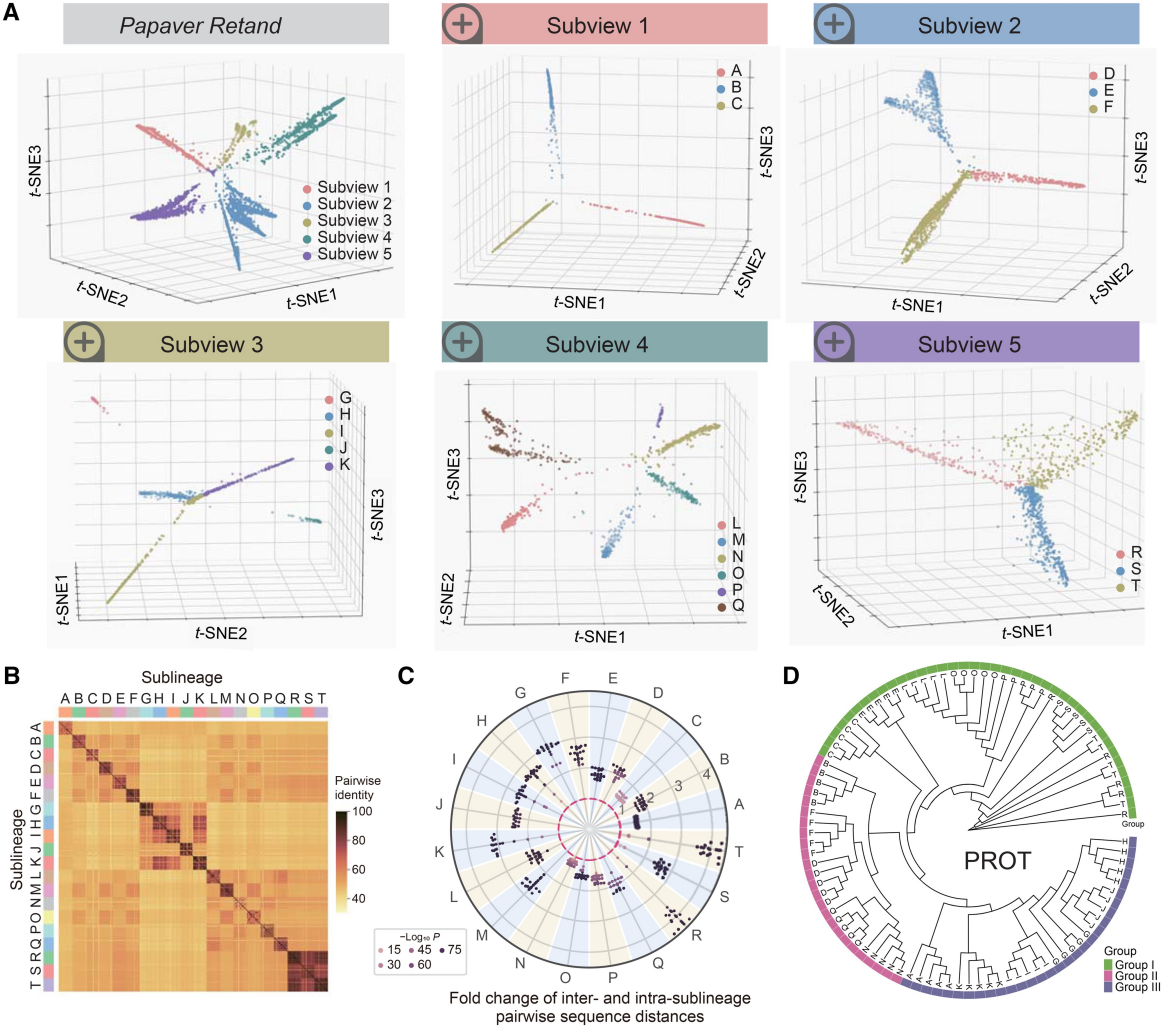
Sublineage clustering by LTR_Stream on ***Retand*** LTR-RTs of the ***Papaver*** group **A**. 3D dot plots showing different sublineages in different subviews. Each dot represents one module sequence. Colors of dots indicate different subviews or sublineages. **B**. Heatmap showing pairwise identity of randomly selected LTR-RTs. **C**. Swarm plot showing fold change of inter- and intra-sublineage pairwise sequence distances. Colors of dots indicate *P* values determined by Wilcoxon rank-sum test. The red dashed circle represents a fold change of 1. **D**. Rootless phylogenetic tree showing randomly sampled PROT sequences from the 19 sublineages (without sublineage M). The outermost color blocks represent different groups.

Having confirmed LTR_Stream’s effectiveness with simulation, we next evaluated LTR_Stream on real LTR-RTs from two closely related plant groups (the *Papaver* group and *Gossypium* group) (Table S2). We classified LTR-RTs from each of these two closely related groups into LTR lineages (Tables S3 and S4) and selected six of them (*Retand*, *Ale*, *CRM*, *Ogre*, and *Tork* of the *Papaver* group and *Tekay* of the *Gossypium* group) as datasets for evaluation. Notably, the six datasets contain varying numbers of LTR-RTs, ranging from over 10,000 to a few hundred, allowing a comprehensive evaluation of LTR_Stream across different data scales. The results are presented in **Figure 2** and Figures S6–S10, respectively. For example, Figure 2A illustrates sublineage clustering (labeled from A to T) of the *Retand* set at different subviews. To evaluate these sublineages, we randomly selected 20 LTR-RT sequences from each and calculated their pairwise sequence distances (Figure 2B). We found that in most cases (51 of 53), sequences from different sublineages were significantly more divergent than those from the same sublineage (with fold change > 1 in Figure 2C and Figures S6–S10). This indicates a higher degree of nucleotide sequence similarity within each sublineage. In the case of *Retand* LTR-RTs from the *Papaver* group, to further validate sublineage clustering of LTR_Stream, we utilized their conserved protein domains to construct phylogenetic trees [19,22]. Given the significant presence of nonautonomous LTR-RTs lacking conserved protein domains (Figure S11), we first constructed an evolutionary tree using the most widely distributed PROT domain across various sublineages. The tree confirmed most (16/19) of the sublineages (Figure 2D). For the sublineages R, T, and S that exhibit clustering confusion on the tree, we found significant differences in their nucleotide sequences (Figure S12), validating the rationale behind LTR_Stream clustering. To validate the clustering of sublineage M, which only possesses the GAG domain, we also constructed an evolutionary tree using this domain (Figure S13A). The tree showed that sublineage M clusters with sublineage L, with the main difference being that L contains additional PROT domain (Figures S11 and S13B). Based on these findings, we proposed an evolutionary model of *Retand* LTR-RTs in the three *Papaver* species (Figure S13B). This model is also supported by phylogenetic trees constructed from the conserved protein domains of PROT (Figure S13C) and GAG (Figure S13D) within each sublineage. In summary, these analyses confirm the reliability of LTR_Stream and show that nucleotide sequence clustering with LTR_Stream can achieve higher resolution than clustering based on conserved protein domains. Additionally, the estimated insertion time of different sublineages varied, suggesting the reliability of the results (Figure 2C, Figures S6–S10).

### Sublineage-level LTR-RT clustering reveals differential LTR-RT distributions between subgenomes

**Figure 3:**
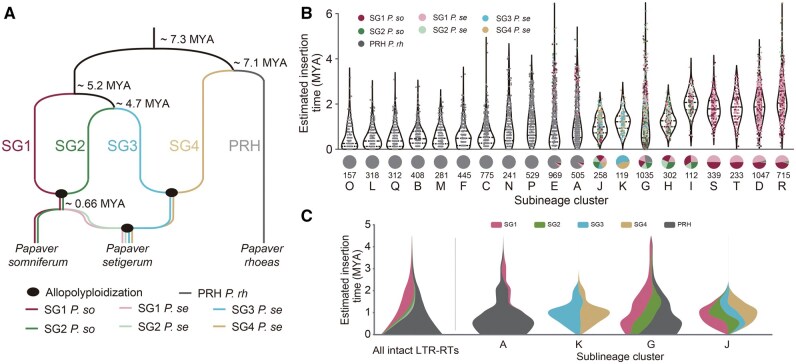
Distribution of 20 sublineages of ***Retand*** LTR-RTs across the five ***Papaver*** subgenomes **A**. Species phylogenetic tree of three *Papaver* species. The divergence times (MYA) between subgenomes are indicated on the tree (not to scale). Black ellipses represent allopolyploidization events. Lines in different colors represent different subgenomes. **B**. Top: violin plot showing the distributions of the estimated insertion time for each sublineage. Colors of dots represent different subgenomes. Bottom: pie chart illustrating the proportions of different sublineages in each subgenome. The number of intact LTR-RTs of each sublineage is indicated below the pie charts. **C**. Theme river plot illustrating the dynamic changes of LTR-RTs over different time periods across the five subgenomes. The far-left panel shows the overall dynamic changes of LTR-RTs across the five subgenomes, while the four panels on the right display the dynamic changes of LTR-RTs in each of the four sublineages. MYA, million years ago; *P*. *so*, *Papaver somniferum*; *P*. *se*, *Papaver setigerum*; *P*. *rh*, *Papaver rhoeas*.

The clustering of LTR-RTs at a finer sublineage level using LTR_Stream provides a novel perspective for studying and comparing closely related genomes/subgenomes. We performed an in-depth analysis of the distribution of *Retand* LTR-RTs across different genomes/subgenomes in three *Papaver* species (*P*. *rh*, *P*. *so*, and *P*. *se*) [13]. As shown in **Figure 3**A, these three species underwent complex differentiation and allopolyploidization events. Historically, five subgenomes (SG1–SG4 and PRH) have been identified [23], with PRH diverging early [∼ 7.1 million years ago (MYA)] from the other four subgenomes and evolving independently into the later *P*. *rh*. In contrast, SG1 and SG2, as well as SG3 and SG4, merged after their differentiation, leading to new species, with the former being the ancestor of *P*. *so*, and the latter not yet identified in existing species. These two species, each containing two subgenomes, later merged and evolved into *P*. *se*. Therefore, *P*. *rh*, *P*. *so*, and *P*. *se* each contain one, two, and four subgenomes, respectively. Due to the relatively short divergence time (∼ 0.66 MYA), SG1 and SG2 in *P*. *se* are highly similar to those in *P*. *so*.

We found that LTR_Stream effectively reveals the differential distribution of LTR-RTs across the five subgenomes. As shown in the pie charts of Figure 3B, we observed that 11 sublineages are predominantly distributed in the PRH, four are mainly found in SG1 (with similar quantities in SG1 *P. se* and SG1 *P. so*), and five are shared across more than one subgenome. Analysis of insertion times indicates that sublineages predominantly located in PRH have the most recent insertion times, suggesting that LTR-RTs in PRH are currently more active than those in the other four subgenomes. Figure 3C illustrates the evolutionary patterns of sublineages across different subgenomes. For example, sublineage A was present in both PRH and SG1 before 2 MYA, but it subsequently underwent an expansion in PRH while nearly disappearing in SG1. At the same time, we observed sublineages (such as K, G, and J shown in Figure 3C) that expanded almost simultaneously in multiple subgenomes. This reflects the differing adaptive strategies of these sublineages in their long-standing evolutionary arms race [24] with the host genomes.

### Sublineage-level LTR-RT clustering demonstrates spread of autonomous *Retand* LTR-RTs between species like retroviruses

**Figure 4:**
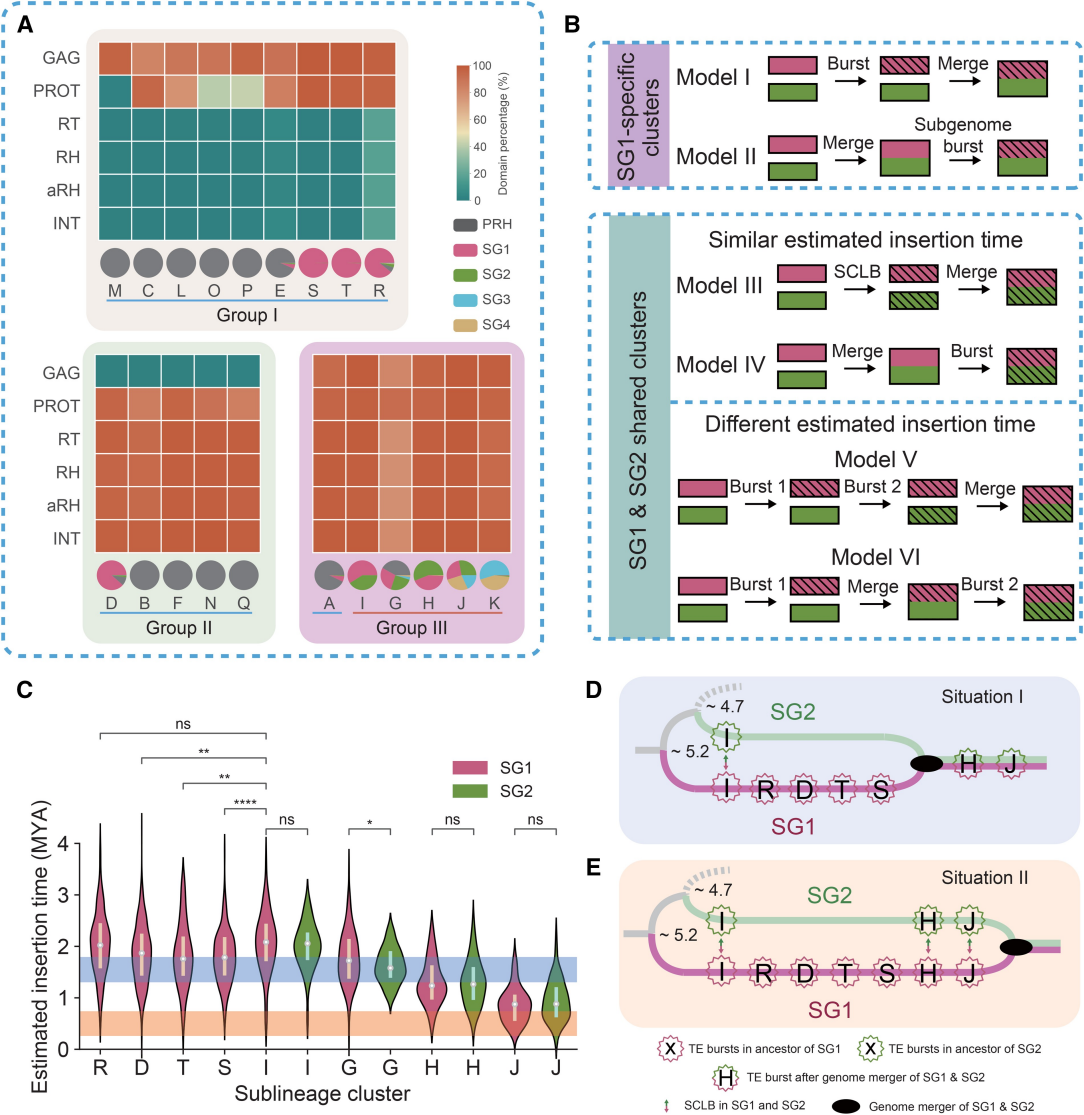
Using sublineages as molecular markers to investigate the spread patterns of LTR-RTs in the progenitors of different ***Papaver*** subgenomes **A**. Heatmap showing protein domain percentages of the 20 clustered sublineages by LTR_Stream. The pie chart at the bottom shows the distribution across different subgenomes (SG1 *P. so* and SG1 *P. se* were merged into SG1, and SG2 *P. so* and SG2 *P. se* were merged into SG2). **B**. Possible models to explain SG1-specific (top) and SG1 & SG2 shared (bottom) sublineage bursts. Two subgenomes are denoted with different colors. The shading represents LTR-RT bursts. **C**. Violin plot showing estimated insertion time of SG1- and SG2-specific sublineages. Colors of violins indicate in which subgenome (SG1 or SG2) LTR-RTs are. The orange shadow denotes the previously reported allopolyploidization time, while the blue shadow denotes another possible time that we proposed. Wilcoxon rank-sum test results are also annotated (ns, *P* > 0.05; *, 0.01 < *P* ≤ 0.05; **, 0.001 < *P* ≤ 0.01; ***, 0.0001 < *P* ≤ 0.001; ****, *P* ≤ 0.0001). **D**. and **E**. Two possible sublineage burst situations in SG1 and SG2. Colors of lines represent the corresponding subgenomes. Circles denote LTR-RT sublineage bursts. Panels D and E are for illustrative purposes only and does not adhere to a strict scale. SCLB, simultaneously cross-species LTR-RT burst; TE, transposable element; ns, not significant.

The sublineages clustered by LTR_Stream in different subgenomes can serve as molecular markers for host genome evolution and also provide insights into how LTR-RTs spread and evolve across the three *Papaver* species. Based on the proportion of conserved protein domains, we classified these sublineages into three groups (**Figure 4**A). Sublineages of Group I mainly contain the GAG and PROT domains, those of Group II lack the GAG domain, and those of Group III include all six domains. For Group II, to rule out potential annotation loss due to divergence between GAG sequences and the database, we confirmed the distance from the 5′ LTR to the start of the PROT open reading frame (ORF) and found that this distance is significantly shorter in Group II sublineages compared to the other two groups (Figure S14). Groups I and II, lacking key protein domains, are non-autonomous LTR-RTs, while most in Group III are autonomous LTR-RTs. The phylogenetic trees constructed based on the consensus sequences of GAG and PROT (Table S5) for each sublineage suggest that sublineages in Group I likely shared a protein domain loss event (Group II as well) (Figure S13). Due to the recent divergence time (Figure 3A) [23] and similar transposon distributions of SG1 *P. so* and SG1 *P. se* (also SG2 *P. so* and SG2 *P. se*) (Figure 3B), we assume that they share most transposon insertions and do not distinguish them in this section. The pie chart in Figure 4A shows the subgenome distribution of these 20 sublineages. Sublineages with > 80% distribution in a specific subgenome are defined as SG-specific sublineages (including 15 sublineages, annotated with blue line in Figure 4A), while the remaining five (sublineages I, G, H, J, and K, annotated with red line in Figure 4A) are SG-shared sublineages. We found that all of the five SG-shared sublineages belong to Group III, indicating a broader distribution of autonomous LTR-RTs in the three species.

Both SG-specific and SG-shared sublineages are important molecular markers. For those sublineages that burst after the subgenome progenitors diverged from their common ancestor, SG-specific bursts typically accumulate during periods of independent evolution of progenitors of subgenomes (Model I in Figure 4B), while the SG-shared sublineages may exhibit various scenarios (Models III–VI in Figure 4B). For those sublineages with similar estimated insertion times in two subgenomes, we proposed two possible models: (1) the progenitors of the two subgenomes independently experienced simultaneously cross-species LTR-RT bursts (SCLBs) followed by allopolyploidization (Model III in Figure 4B); (2) the sublineage burst occurred after allopolyploidization (Model IV in Figure 4B).

For those sublineages with different insertion times, we also proposed two models: (1) the sublineages burst in the progenitor of one subgenome and were then horizontally transferred to the other (Model V in Figure 4B); (2) the sublineages burst in the progenitor of one subgenome and then, after allopolyploidization, underwent another burst across the entire genome (Model VI in Figure 4B).

Taking the sublineages mainly distributed in SG1 and SG2 as an example, we found that sublineages T, R, S, and D were specific to SG1, while sublineages I, G, H, and J were shared between SG1 and SG2 (Figure 4A). The estimated insertion time showed that all of these sublineages burst after the progenitors of SG1 and SG2 diverged from their common ancestors (Figure 4C). For SG-shared sublineages, excepting sublineage G, sublineages I, H, and J exhibited similar estimated insertion time across the two subgenomes (Figure 4C). Sublineage I was distinct in that it burst before either of the SG1-specific sublineages such as D, T, and S. We considered that if sublineage I burst after the allopolyploidization (Model IV), then sublineages D, T, and S should more closely resemble Model II — however, we thought this was less likely because it did not conform to the current understanding of retrotransposon mechanisms. Hence, we inferred that sublineage I reflected an SCLB event in progenitors of SG1 and SG2 (Model III). Such an SCLB event was intriguing, because it indicated that horizontal transfer of LTR-RTs [25,26] between closely related species and the subsequent expansions in host genome could occur very rapidly, more akin to a retrovirus pandemic. For sublineages H and J, it was hard to say whether they followed Model III or IV, because there were no subsequent SG1-specific or SG2-specific sublineage bursts. If H and J followed Model IV, then allopolyploidization was estimated to have occurred 1.30–1.79 MYA (that is, the median estimated insertion time between H and T, shown as the blue shadow in Figure 4C) and so we proposed a possible sublineage burst scenario (Figure 4D). However, if H and J followed Model III, the allopolyploidization event should have occurred later (Figure 4E), which is consistent with a previous report (the estimated time was 0.26–0.74 MYA, shown as an orange shadow in Figure 4C) [23]. The latter hypothesis implied at least three SCLB events (including sublineages I, H, and J) in the progenitors of SG1 and SG2, very strong evidence proving that *Retand* could spread between species like retroviruses. Interestingly, sublineages I, H, and J all belong to Group III, the autonomous LTR-RT group, suggesting that compared to non-autonomous LTR-RTs, these autonomous elements have a greater potential for cross-species transmission. Regardless of which evolutionary scenario depicted in Figure 4D or E is correct, sublineage I in SG1 and SG2 serves as a strong molecular marker for a past SCLB event, suggesting that historically, *Retand* LTR-RTs could spread across species in a manner similar to retroviruses.

### Modules identified by LTR_Stream reveal the diverse activity of LTR-RTs across host subgenomes

During the long-term arms race between host genomes and LTR-RTs, host genomes employ various mechanisms to eliminate LTR-RTs, thereby generating truncated fragments of LTR-RTs [27]. Compared to intact LTR-RTs with paired LTRs, these truncated fragments are more numerous and widely distributed across the genome. Recent evidence suggests that these truncated fragments can influence genome functions, such as transcriptional regulation [28]. Luckily, modules identified by LTR_Stream during the clustering process, which represent homologous nucleotide sequence fragments shared among certain LTR-RTs, facilitate the genome-wide identification and differentiation of these truncated fragments.

**Figure 5:**
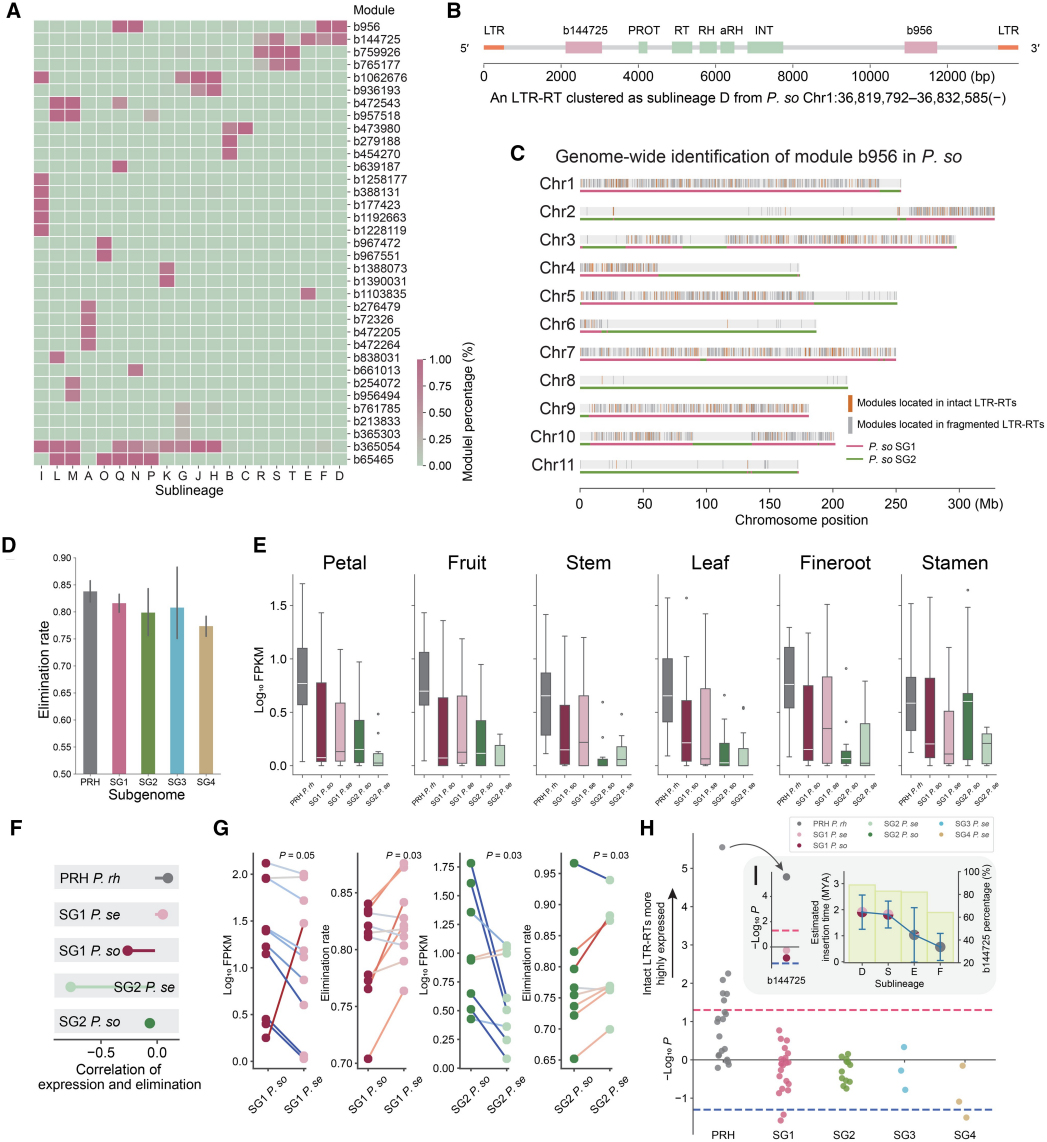
The activity diversity of ***Retand*** LTR-RTs across different ***Papaver*** subgenomes at the module level **A**. Heatmap illustrating the distribution of different modules across different sublineages. The numbering starting with “b” represents the module ID. **B**. An example showing the position of modules on an LTR-RT sequence. The light green rectangles represent the conserved protein ORFs. The pink rectangles represent the modules identified by LTR_Stream. **C**. Using module b956 as an example, its distribution on the *P. so* genome is shown. The modules located in intact LTR-RT regions are marked in orange, while those located in truncated fragments are highlighted in gray. The two subgenomes are indicated by lines in different colors. **D**. Bar chart displaying the elimination rate of LTR-RTs in different subgenomes, assessed using modules. **E**. Box plots showing the expression levels of modules in PRH, SG1 *P*. *so*, SG1 *P*. *se*, SG2 *P*. *so*, and SG2 *P*. *se* across six tissues. **F**. Lollipop chart illustrating the correlation between module expression levels and elimination rates in different subgenomes. **G**. Dumbbell plots showing the changes in expression levels and elimination rates of *Retand* LTR-RTs before and after the last allopolyploidization in *P*. *se*. **H**. Swarm plot showing the expression level differences between intact LTR-RTs and truncated fragments across the five subgenomes. The red dashed line represents a higher expression in intact LTR-RTs with a *P* value of 0.05 (Wilcoxon rank-sum test). The blue dashed line represents a higher expression in truncated fragments with a *P* value of 0.05 (Wilcoxon rank-sum test). **I**. Using module b144725 as an example, the plot showing its preferential expression between intact LTR-RTs and truncated fragments across different subgenomes, alongside the activity level of the corresponding sublineage in the respective subgenomes. ORF, open reading frame; FPKM, fragments per kilobase of transcript per million mapped reads.

From *Retand* LTR-RTs of three *Papaver* species, we filtered 35 modules that showed differential distributions among sublineages and exhibited low reciprocal overlap rates (< 0.5) (**Figure 5**A). Some modules were sublineage-specific, such as module b956494 (the sixth row from bottom in Figure 5A), which was predominantly distributed in sublineage M (the third column from left in Figure 5A). In contrast, some modules were shared by several sublineages. For example, b956 (the first row from top in Figure 5A) was mainly shared by sublineages Q, N, F, and D. These modules intrinsically reflect sublineage-specific nucleotide sequences. For instance, Figure 5B illustrates the positional annotations of two sublineage D-associated modules, b144725 and b956, within a single LTR-RT sequence. These modules can be used to search for homologous sequences across the whole genome. For example, Figure 5C shows the positional annotation of module b956 identified in *P*. *so*. The module b956 is predominantly distributed in sublineages F, N, Q, and D. The first three sublineages are primarily distributed in PRH, whereas sublineage D is predominantly distributed in SG1 (Figure 3B). Consequently, b956 is also observed to be mainly located in SG1 in *P. so* (Figure 5C). By calculating the proportion of these modules within intact LTR-RTs, we estimated the elimination rate of LTR-RTs in subgenomes. Interestingly, we found that PRH, within which *Retand* is most active, also exhibits the highest elimination rate (Figure 5D). Using transcriptome data from six tissues (including petal, fruit, stem, leaf, fineroot, and stamen) [13] across the three species, we evaluated the transcriptional activity of these modules. Modules in PRH were consistently found to have the highest expression levels across all six tissues (Figure 5E). Among these tissues, the stamen, as a reproductive organ, may experience reduced methylation due to epigenetic reprogramming, thereby reactivating some LTR-RTs [29]. However, increased transposon expression was only observed in SG2 (including both SG2 *P*. *so* and SG2 *P*. *se*) (Figure 5E), suggesting that different subgenomes exhibit varying capacities to suppress transposons following a reduction in methylation. *Retand* in SG2, which is more active in reproductive organs, may be more prone to hereditary accumulation. Previous studies have reported that more active transposons often exhibit higher elimination rates [24]. To investigate the correlation between activity and elimination at the module level, we analyzed their relationship across all five subgenomes but found no strong positive correlation (Figure 5F). This indicates that different modules exhibit relatively dynamic changes within each subgenome. For instance, modules with higher expression levels and lower elimination rates tend to be in an expansion phase compared to others. Since SG1 and SG2 in *P*. *so* and *P*. *se* diverged relatively recently (∼ 0.66 MYA) [23], and the latter underwent an additional allopolyploidization (Figure 3A), these genomes provide an excellent system to study the impact of allopolyploidization on transposon activity. Our findings reveal that most modules across both subgenomes show a general decline in overall transcription levels accompanied by an increase in elimination rates (Figure 5G). While previous studies have reported that allopolyploidization can induce genome shock, resulting in transposon activation [30–34], the opposite pattern is observed in *P*. *se*. Next, we compared the expression levels of fragmented modules and those located within intact LTR-RTs. Indeed, we would expect to observe a higher expression tendency in those within intact LTR-RTs, as fragmented LTR-RTs have already lost their transposition activity. Surprisingly, in SG1 and SG4, we found that the expression of fragmented LTR-RTs of three modules was even higher, which raises questions about the potential effects of high expression of fragmented transposons on their transposition behavior (Figure 5H). Overall, modules in the more active PRH subgenome tend to show higher expression of intact LTR-RTs (Figure 5H), suggesting that the expression of intact LTR-RTs plays a more significant role in transposon activity. For instance, we observed that the expression of b144725 is significantly higher in intact LTR-RTs in PRH, while in SG1, there is no significant difference in expression between fragmented and intact LTR-RTs (Figure 5I). Module b144725-associated sublineages D and S have an earlier insertion time in SG1, while sublineages E and F were inserted later in PRH (Figure 5I), indicating that they still maintain considerable activity in PRH. Our analysis demonstrates that the modules identified by LTR_Stream provide a more comprehensive understanding of the diversity of LTR-RT activity across different subgenomes, offering a more nuanced perspective for studying the arms race between LTR-RTs and the host genome.

### Modules identified by LTR_Stream influence nearby TAD-like boundaries

**Figure 6:**
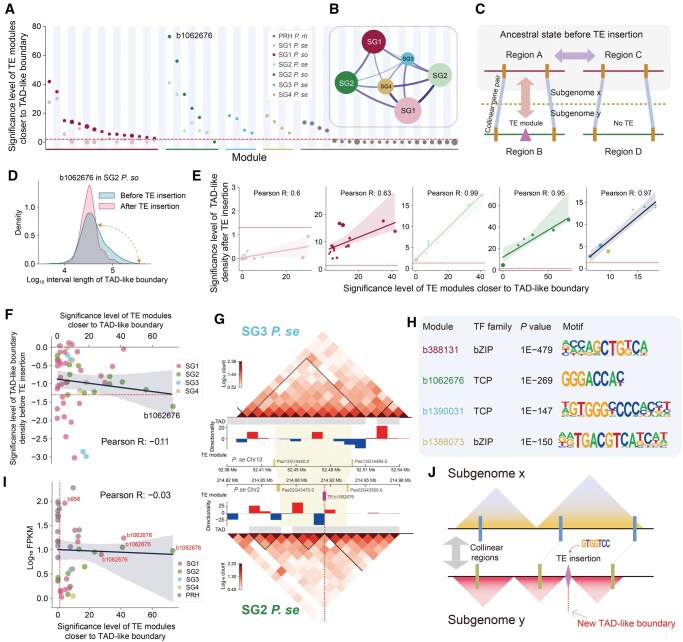
Modules carrying certain TCP-related or bZIP-related motifs may mediate the formation of new TAD-like boundaries **A**. Dot plot showing the significance of proximity between modules and TAD-like boundaries (Wilcoxon rank-sum test). Colors of dots represent different subgenomes. **B**. Graph showing subgenomes that used to identify colinear synteny gene pairs. The number of colinear synteny gene pairs is represented by the width of the edges. **C**. Schematic diagram illustrating how to compare changes in TAD-like structures after LTR-RT insertion. **D**. Density plot showing changes in adjacent TAD-like boundary intervals around module b1062676 after LTR-RT insertion. **E**. Dot plots showing the association between the significance of TAD-like boundary density changes near different modules after LTR-RT insertion (Y-axis) and the significance of modules closer to TAD-like boundaries (X-axis) across different subgenomes (Wilcoxon rank-sum test). The red dashed line represents a *P* value of 0.05 for the significance of 0.05. **F**. Dot plot showing the significance of TAD-like boundary density near sites with LTR-RT insertions but before insertion compared to sites without any LTR-RT insertions, along with the significance of modules closer to TAD-like boundaries (Wilcoxon rank-sum test). Colors of dots represent different subgenomes. **G**. An example illustrating the changes in TAD-like structures after LTR-RT insertion in SG2 *P*. *se* (bottom), compared with that in SG3 *P*. *se* (top). The heatmaps show Hi-C interactions at a 10-kb resolution. The bar charts show the directionality index. The purple rectangle represents module b1062676. The yellow rectangles represent colinear synteny gene pairs identified by MCScanX [36]. **H**. Most enriched TCP-related and bZIP-related motifs in the four modules from different subgenomes. Colors of module IDs represent corresponding subgenomes. **I**. Dot plot showing module total expression level (Y-axis) and its correlation to the significance of modules closer to TAD-like boundaries (X-axis). Colors of dots represent different subgenomes. **J**. A model schematic illustrating that modules carrying TCP-related and bZIP-related motifs might mediate the formation of new TAD-like boundaries. TAD, topologically associating domain; Hi-C, high-throughput chromosome conformation capture.

Next, we explored the functional impact of the modules identified by LTR_Stream on the genome. Previous studies in wheat have reported that LTR-RTs are involved in higher-order chromatin organization [35]. Using high-throughput chromosome conformation capture (Hi-C) sequencing data, we analyzed the potential associations between these modules and higher-order chromatin organization across the genome. We discovered that certain modules are closer to TAD-like boundaries compared to random regions (**Figure 6**A), such as module b1062676 in SG2 *P. so*, which we define as near-TAD modules. We proposed two possible explanations for this phenomenon: (1) these near-TAD modules contribute to the generation of nearby TAD-like boundaries, or (2) LTR-RTs carrying these near-TAD modules are preferentially inserted near TAD-like boundaries. To determine which of these scenarios better explains this intriguing observation, we used colinear synteny gene pairs [36] across different subgenomes as a framework to identify homologous region pairs (Figure 6B and C). Due to the extensive transposon bursts and accumulation in PRH, we excluded it from this analysis. In detail, we first selected homologous region pairs that contain one region A (without any near-TAD modules) and one region B (containing near-TAD modules) (Figure 6C). We hypothesized that the TAD-like structures in region A are not influenced by LTR-RTs and therefore represent a state closer to the ancestor compared to region B. We then compared the TAD-like boundary density between these region pairs. We found that the distance between adjacent TAD-like boundaries became shorter after TE insertion, indicating that this higher TAD-like boundary density was caused by TE. Figure 6D shows an example of module b1062676 in SG2 *P*. *so*. In general, we found that, except for SG1 *P*. *se*, the region A-associated TAD-like boundary density was significantly lower than that of region B across all other subgenomes (Figure 6E). Moreover, the significance of this reduction was strongly positively correlated with the significance of modules closer to TAD-like boundaries, with correlation coefficients exceeding 0.95 in SG2, SG3, and SG4 (Figure 6E). These findings support the first hypothesis: LTR-RT insertions into subgenomes indeed influence the nearby TAD-like structures. Next, we selected region pairs unaffected by any LTR-RT insertions (regions C and D in Figure 6C) as negative controls for region A. If the second hypothesis were correct, we would expect the region A-associated TAD-like boundary density to be significantly higher than that in regions C and D. However, as shown in Figure 6F, this was not the case. On the contrary, we observed that the TAD-like boundary density near region A was even lower in certain cases, such as those involving modules in SG1 and SG3 (Figure 6F). Additionally, the significance was not positively correlated with the significance of modules closer to TAD-like boundaries (Figure 6F), further disproving the second hypothesis. Thus, we conclude that certain near-TAD modules indeed induce changes in TAD-like structures. Figure 6G illustrates an example: in homologous pair regions across SG3 and SG2, the insertion of the LTR-RT module b1062676 in SG2 may contribute to the formation of a new TAD-like boundary nearby (indicated by the red dashed line), which is absent in SG3. Notably, compared to SG1 and SG2 in *P*. *se* (light red and light green in Figure 6A), the proximity significance of modules to TAD-like boundaries was higher in *P*. *so* (dark red and dark green). This observation suggests that allopolyploidization events may attenuate the impact of modules on TAD-like boundary formation. Previous studies in plants have shown that the bZIP and TCP transcription factor families can influence 3D chromatin structures and their associated motifs are enriched near TAD-like boundaries [37,38]. Consistently, we identified that motifs associated with these transcription factors were enriched within near-TAD modules (Figure 6H; File S1). Given that these motifs were also enriched near TAD-like boundaries in the corresponding subgenomes (File S2), we proposed that the presence of TCP- and bZIP-associated motifs in these modules is likely the mechanism by which they influence TAD-like structural organization. We observed that modules with higher proximity significance to TAD-like boundaries also shared more TCP-related and bZIP-related motifs with each other (Figure S15). Transcriptomic analysis revealed that b1062676 in SG1 and SG2 maintains a certain level of activity [fragments per kilobase of transcript per million mapped reads (FPKM) around 10] (Figure 6I), indicating its potential to continuously influence the higher-order 3D structure of its host genome. Based on these findings, we proposed a model (Figure 6J), that is, modules such as b1062676 which carry bZIP-related and TCP-related motifs may mediate the formation of new TAD-like boundaries in their vicinity.

## Discussion

The diversity and widespread distribution of LTR-RTs in plants make them valuable molecular markers for genomic evolution. For example, as early as a decade ago, researchers used polymerase chain reaction (PCR)-based TE markers to reveal phylogenetic relationships within and between plant species [39]. In recent years, with advances in sequencing technologies, *k*-mer analysis based on assembled genomes has been employed for LTR-RT marker identification [40,41]. Typically, after the divergence of ancestral species, subgenome-specific TEs may accumulate during independent evolution, while allopolyploidization events halt this biased accumulation. By comparing the divergence time of subgenome ancestors and the insertion time of subgenome-specific LTR-RTs, these methods can identify allopolyploidization events and estimate when they occurred. The high-resolution LTR-RT clustering achieved by LTR_Stream facilitates in-depth analysis of LTR-RT burst and propagation patterns in closely related species. Take three *Papaver* species as an example, we observed that the ancestors of two subgenomes experienced simultaneous bursts of the same sublineage during their independent evolution following species divergence, with such events likely occurring three times in the recent four million years. Although horizontal transfer of LTR-RTs among species is not uncommon, the near-simultaneous bursts of same LTR-RT sublineages in closely related species was rarely reported. These SCLBs suggest that these sublineages may have once possessed the ability to spread rapidly between species, akin to plant viruses. However, the precise mechanisms underlying the cross-species transmission of *Retand* transposons remain unclear. On one hand, *Retand* lacks the ENV protein, which is essential for interacting with cell surface receptors and mediating fusion. On the other hand, the plant cell wall poses a physical barrier to intercellular LTR-RT transmission. We found that all sublineages potentially associated with SCLBs are autonomous and identified two additional ORFs encoding transmembrane proteins in *Retand* (Figure S16), which may functionally substitute for ENV proteins. These findings provide valuable insights for further research about mechanisms of LTR-RT cross-species transmission. Additionally, further exploration of how host genomes counteract such cross-species transmission threats is warranted, as it may offer critical references for addressing potential plant viral threats in the future.

In addition to serving as molecular markers, the insertion of TEs can directly or indirectly influence plant genome function. For example, numerous studies have reported that TEs carrying specific *cis*-regulatory motifs can affect the expression of nearby genes, thereby reshaping gene regulatory networks [7,42]. In recent years, the impact of TEs on higher-order chromatin structures has also been increasingly documented in plants [43]. For instance, a study in cotton has reported that, TEs are linked to transitions between active and inactive chromatin regions, and species-specific TE expansions may mediate the formation of species-specific TAD boundaries [44,45]. However, the specific TEs involved and their underlying mechanisms remain unclear. Leveraging high-resolution clustering results from LTR_Stream, we compared syntenic gene modules and found that the insertion of certain LTR-RT modules correlates with a significant increase in TAD boundary density in their vicinity. Further analysis revealed that these modules are enriched with binding motifs for TCP and bZIP transcription factors. Given that TCP and bZIP motifs are also enriched at TAD boundaries in plants such as rice [37] and cotton [45], we hypothesize that TE-introduced motifs may facilitate the formation of new TAD boundaries in adjacent regions. Although these transcription factors are considered potential regulators of TADs in plants, the specific insulators mediating TAD formation remain unidentified, and further research is needed to elucidate the underlying mechanisms.

TEs and polyploidization are considered two major driving forces in plant genome evolution [24]. Previously, some hypotheses and evidence have suggested that the genomic shock induced by polyploidization could trigger massive TE expansions [33,46]. However, recent studies have shown that polyploidization does not necessarily lead to TE bursts in many species [33,46]. *P*. *se*, formed through the allopolyploidization of the progenitor of *P*. *so* and an unknown species, provides an ideal system to study TE dynamics under polyploidization. Using high-resolution clustering results from LTR_Stream, we found that, compared to *P*. *so*, the potential threat of *Retand* to the genome of *P*. *se* is significantly reduced, evidenced by lower expression levels and higher elimination rates. Notably, we observed a weakened association between TE modules and TAD boundaries after allopolyploidization, although the mechanisms driving this change remain unclear. We noticed that previous studies have shown that polyploidization itself can alter higher-order chromatin structures, such as A/B compartment transitions and TAD reorganization [43]. For example, in soybean, changes in epigenetic modifications (*e.g.*, H3K4me3, H3K9me2, and CG/CHG methylation levels) during polyploidization may drive TAD restructuring [47]. This mechanism parallels findings in animal genomes, where epigenetic changes leading to gains or losses of local transcriptional activity can promote the formation or loss of TAD boundaries [47]. Our study demonstrates that the impact of TEs on the genome is attenuated in *P*. *se* following polyploidization. Furthermore, considering the significantly higher TE activity in *P*. *rh* and *Papaver bracteatum* [48], which have not undergone recent polyploidization, we propose that polyploidization may serve as an adaptive strategy in *Papaver* species to counteract TE expansion. Given that TE loss has also been reported in other polyploid species [33,46], we further speculate that this mechanism may be a widespread phenomenon in plants, which needs further investigation.

## Materials and methods

### LTR-RT representation with module sequences

LTR_Stream clusters LTR-RT DNA sequences by first performing self-BLAST with BLASTN (v2.11.0+) [49]. This comparison helps identify homologous regions within the sequences. LTR_Stream then constructs an undirected graph model based on these comparisons. LTR_Stream segments LTR-RT sequences into different sections and identifies them as distinct modules, representing each original LTR-RT sequence as a module sequence. Specifically, for a given self-BLAST result, LTR_Stream builds an undirected graph *G*. The vertices of *G* represent sections of LTR-RT sequences that are identified homologous to other sequences. For any pair of vertices *v* and *j* in the graph, we add an edge (*v, j*) to *G* if either of the following conditions is met (Figure S1): (1) sections *v* and *j* are identified by BLAST as a homologous pair, and e-value is less than a given threshold; (2) sections *v* and *j* belong to the same LTR-RT, and the mutual overlap of their positions exceeds a given threshold.

Since LTR-RTs often share a lot of similarity, BLAST typically provides a large number of homologous alignment records. To manage this complexity, LTR_Stream limits the total number of alignment records by setting the maximum output records for each LTR-RT. LTR_Stream then efficiently counts the connected components in large graphs using a disjoint set. Vertices belonging to each connected component are then merged into a module (Figure S1). According to the original positions of each module, LTR_Stream represents each LTR-RT as a module sequence. In cases where there are too many modules, LTR_Stream represents only those LTR-RTs with the most widely distributed modules.

### Sublineage-level LTR-RT clustering

LTR_Stream takes the module sequences generated from the previous step and calculates Levenshtein distances [21] between different module sequences and reduces the distance matrix to a 3D space using *t*-distributed stochastic neighbor embedding (*t*-SNE) [50]. Due to the accumulation of mutations, we expect that older LTR-RTs would have fewer identifiable homologous segments compared to more recently inserted LTR-RTs. Consequently, we assume that older LTR-RTs would have shorter module sequences and relatively smaller Levenshtein distances when compared with each other. Hence, older LTR-RTs are expected to cluster closer together near the center, while more recent ones, from different evolutionary branches, would be positioned further out in the 3D space. Next, LTR_Stream calculates the angular differences between different module sequences relative to the center, constructing a new distance matrix. LTR_Stream clusters these with agglomerative clustering [51], optimizing the clustering with ElPiGraph [52] to reconstruct modular sequence trajectories. Part of the code framework references STREAM [53]. In some cases, LTR-RTs might have complex evolutionary histories, making it difficult to group them definitely. For such cases, LTR_Stream focuses on a specific category and refines the clustering iteratively until either the dimensionality reduction no longer fits the assumption or it reaches a predefined maximum iteration depth. This strategy resembles hierarchical clustering, but due to the sparsity of the features extracted by LTR_Stream, we do not guarantee the intermediate hierarchical clustering. To evaluate the final clusters (sublineages), LTR_Stream randomly selects several LTR-RT sequences from each cluster, calculates their pairwise identities, and employs the Wilcoxon rank-sum test to assess whether intra-class similarity significantly exceeds inter-class identity. This helps adjust parameters and refine the clustering if necessary. Finally, LTR_Stream consolidates the final clustering results obtained from different perspectives and saves them in a Tab-Separated Value (TSV) format file. LTR_Stream also outputs the coordinates of the module sequences in the final view, facilitating the filtering out of the lower-confidence samples near the origin for users.

### Data simulation

For a given LTR-RT sequence, we simulated LTR-RT bursts iteratively, introducing random structural mutations (including insertions, deletions, and inversions) at each iteration to construct an evolutionary trajectory. To faithfully simulate LTR-RT evolution under natural conditions, we adopted the model proposed by Drost and Sanchez [54], which incorporates indels that occur along the host genome as well as LTR-RT elimination. To test LTR_Stream, we randomly selected one sequence from each of the three LTR lineages (*Ale*, *CRM*, and *Tork*) in the *G*. *herbaceum* genome as the ancestral sequences, and simulated two to four independent evolutionary paths originating from them.

### Genome-wide LTR-RT identification from closely related species

We downloaded the genome assemblies of three *Papaver* [13] and four *Gossypium* species [14,45], as detailed in Table S2. Then, we ran LTR_FINDER (v1.2) [55] with default parameters and LTRharvest (v1.6.2) [56] with parameters “-minlenltr 100 -maxlenltr 7000 -mintsd 4 -maxtsd 6 -motif TGCA -motifmis 1 -similar 85 -vic 10 -seed 20 -seqids yes” on these assemblies. Further, LTR_retriever (v2.9.0) [57] was used to filter high-quality LTR-RTs and estimate insertion times of LTR-RTs. Nucleotide sequences of LTR-RTs were extracted by BEDTools (v2.30.0) [58], which were then used as input to LTR_Stream. The insertion times of each LTR-RT were estimated by LTR_retriever with synonymous substitutions per site per year considered to be 8.1 × 10^−9^ for the *Papaver* species [13] and 3.5 × 10^−9^ for the *Gossypium* species [14].

### LTR-RT protein domain analysis

LTR-RT protein domains were annotated by DANTE (v1.0.0) [59], using the database viridiplantae_v3.0, downloaded from the REXdb [19]. For evaluating sublineage clustering of LTR_Stream, five amino acid sequences of PROT and GAG were randomly selected from each cluster (50% LTR-RTs near origin will be filtered out). Then, we ran MUSCLE [60] with default parameters on these sequences and constructed phylogenetic trees with FastTree (v2.1.11) [61]. The ggtree (v3.10.0) [62] was used to plot and annotate the two phylogenetic trees.

To clarify the phylogenetic relationships between sublineages, we randomly selected 50 GAG and PROT protein sequences from each sublineage to construct consensus domain sequences. Then, for each protein in each sublineage, we ran MUSCLE [60] with default parameters and generated a consensus sequence with biopython (v1.81). Then, with an amino acid sequence from *Athila* lineage as the outgroup, we used MUSCLE, FastTree, and ggtree to generate a phylogenetic tree (as mentioned previously).

### Genome-wide module identification and expression quantification

Modules with a diffScore more than 0.8 were selected, where diffScore was defined as the range of module percentages of different clusters. For each module, we randomly selected ten sequences as seeds for searching genome-wide homologous regions (BLASTN with parameters “-evalue 1e-5 -outfmt 6 -max_target_seqs 10000”). We retained only homologous regions that are at least 50% of the seed length. Considering that homologous regions identified by different seeds may overlap, we merged these regions and assigned the median of their start and end points to the merged region. Next, considering the potential overlap between modules, we filtered out modules that overlapped with longer modules to minimize redundancy (defined as having a median overlap greater than 0.2). The coordinate information of these non-redundant modules was exported to a gene transfer format (GTF) file.

For module expression quantification, we first used STAR (v2.7.8a) [63] to align RNA sequencing (RNA-seq) data from six tissues (including petal, fruit, stem, leaf, fineroot, and stamen) [13] across three *Papaver* species (including *P. rh*, *P. so*, and *P. se*) to their respective genomes [13]. The parameters for STAR were set as “--outSAMtype BAM SortedByCoordinate --sjdbOverhang 149 --readFilesCommand zcat --outFilterMultimapNmax 1000 --winAnchorMultimapNmax 1000”, according to the recommendations of TEtranscripts [64], aiming to maximize the alignment of reads to repetitive regions. Then, TEcount (v2.2.3) [64] was used to quantify each module, and FPKM was estimated using the method implemented in the countToFPKM R package (https://rdrr.io/cran/countToFPKM/man/fpkm.html) as follows:


$${\mathrm{FPKM}}_{\mathrm{module}}=\frac{\mathrm{Coun}t_{\mathrm{module}}}{\mathrm{MedianLengt}h_{\mathrm{module}\;}\times \;\mathrm{TotalCount}}\times 1e9$$

### 3D genome analysis

Hi-C data of *P*. *so* and *P*. *se* were downloaded from the National Center for Biotechnology Information (NCBI) Sequence Read Archive (SRA: PRJNA720042) [13]. Juicer (v1.5.7) [65] with parameter “-s DpnII” was used for counting interactions. TAD-like structure was called by directionality index (DI) method [66]. For each module, we calculated distances of homologous regions to the closest TAD-like boundary and assessed significance against randomly generated regions (of the same number and with the same length) using the Wilcoxon rank-sum test. Colinear synteny gene pairs were identified by MCScanX [36] across different subgenomes. TCP and bZIP motifs were downloaded from the PlantTFDB database [67]. Motif enrichment was performed by HOMER [68]. For each module, an enrichment analysis was performed by comparing module sequences to 3000 randomly selected regions (with median length of module sequences) of the corresponding subgenome. For TAD-like boundaries, an enrichment analysis was performed by comparing their flanking 10-kb regions to 5000 randomly selected regions of the corresponding subgenome.

### Parameter description and adjustment guideline

User-specific datasets require appropriate parameter adjustments to achieve optimal clustering results. LTR_Stream’s parameters are divided into three main categories. The first category includes minOverlap and topModNum, which are used for identifying and selecting modules. The second category involves *t*-SNE-related parameters for dimensionality reduction, such as perplexity, earlyExaggeration, and learningRate. The third category includes parameters used in ElPiGraph. Among these, minOverlap, topModNum, and perplexity are particularly sensitive to the dataset and significantly impact clustering results. To handle this, LTR_Stream provides visualizations of intermediate results to assist users in tuning these three parameters.

minOverlap controls the merging of nearby modules, with a smaller value leading to more merging. topModNum defines the number of most frequent modules selected. Before clustering, LTR_Stream provides the percentage coverage of LTR-RTs by different modular sequence lengths under different topModNum settings. A too small minOverlap merges more modules, causing information loss and generally resulting in shorter module sequences (Figure S17A). We recommend that the difference between the coverage at a modular sequence length of 1 (near the saturation point) and that at length of 10 should not exceed twofold (Figure S17B). A too large minOverlap value results in a jagged increase in the coverage curve due to nested modules, potentially introducing outliers (Figure S17C). Ideally, the curve should rise as smoothly as possible (Figure S17B). We recommend setting minOverlap between 0.80 and 0.99. For topModNum, we suggest a value slightly above the saturation point of coverage for modular sequences of length 2 or 3. If outliers affecting clustering arise, reducing topModNum may help resolve the issue.

For the *t*-SNE perplexity parameter, LTR_Stream outputs the dimensionality reduction results before clustering. A too low perplexity value leads to overly dense local structures (Figure S17D), while a too high value results in overly dispersed clustering (Figure S17E). The ideal perplexity setting produces clustering results as shown in Figure S17F. We recommend an adjustment range of 50–500, with each adjustment changing the value by at least 5% of the current setting. For other parameters, please refer to Table S6 and adjust them as needed.

## Code availability

LTR_Stream is freely available to non-commercial users at GitHub (https://github.com/xjtu-omics/LTR_Stream). It has also been submitted to BioCode at the National Genomics Data Center (NGDC), China National Center for Bioinformation (CNCB) (BioCode: BT007914), which is publicly accessible at https://ngdc.cncb.ac.cn/biocode/tool/BT007914.

## CRediT author statement

**Tun Xu:** Formal analysis, Methodology, Investigation, Software, Writing – original draft. **Stephen J. Bush:** Writing – review & editing. **Yizhuo Che:** Formal analysis. **Huanhuan Zhao:** Software. **Tingjie Wang:** Formal analysis. **Peng Jia:** Software. **Songbo Wang:** Formal analysis. **Peisen Sun:** Formal analysis. **Pengyu Zhang:** Formal analysis. **Shenghan Gao:** Formal analysis. **Yu Xu:** Formal analysis. **Chengyao Wang:** Visualization. **Ningxin Dang:** Visualization. **Yong E. Zhang:** Writing – review & editing. **Xiaofei Yang:** Conceptualization, Writing – review & editing, Funding acquisition, Methodology. **Kai Ye:** Conceptualization, Writing – review & editing, Funding acquisition, Supervision. All authors have read and approved the final manuscript.

## Competing interests

The authors have declared no competing interests.

## Supplementary Material

qzaf061_Supplementary_Data

## ORCID

0000-0003-3194-1834 (Tun Xu)

0000-0001-9341-2562 (Stephen J. Bush)

0000-0003-4483-6582 (Yizhuo Che)

0009-0003-8641-6487 (Huanhuan Zhao)

0000-0001-9238-0968 (Tingjie Wang)

0000-0002-3429-919X (Peng Jia)

0000-0003-4482-8128 (Songbo Wang)

0000-0003-0796-2133 (Peisen Sun)

0000-0001-8696-4983 (Pengyu Zhang)

0000-0002-3810-6527 (Shenghan Gao)

0000-0003-3386-1379 (Yu Xu)

0000-0002-0362-4498 (Chengyao Wang)

0000-0003-2473-1142 (Ningxin Dang)

0000-0003-3770-2383 (Yong E. Zhang)

0000-0002-5118-7755 (Xiaofei Yang)

0000-0002-2851-6741 (Kai Ye)
