# Supplementary material for: Deciphering Complex Interactions Between LTR Retrotransposons and Three Papaver Species Using LTR_Stream
Source: Genomics Proteomics Bioinformatics. 2025 Jul 8;23(4):qzaf061. doi: 10.1093/gpbjnl/qzaf061 (PMC12582370; doi:10.1093/gpbjnl/qzaf061)
Supplement: qzaf061_Supplementary_Data [file qzaf061_supplementary_data.zip › Table S6.docx]

**Table S6 Parameter recommendation**

| **Parameter** | **Recommended value** |
| --- | --- |
| **minOverLapForNovelModule** | **0.80–0.99** |
| **topModNum** | **50–500** |
| blastEvalue | 1E−10 |
| tsneLearningRate | 6 |
| **tsnePerplexity** | **50–500** |
| tsneEarlyExaggeration | 6–8 |
| cluCentCut | 0.1–0.2 |
| maxZoomInLevel | 1–5 |

*Note*: Parameters that significantly influence clustering were highlighted in bold.
