# Supplementary material for: Deciphering Complex Interactions Between LTR Retrotransposons and Three Papaver Species Using LTR_Stream
Source: Genomics Proteomics Bioinformatics. 2025 Jul 8;23(4):qzaf061. doi: 10.1093/gpbjnl/qzaf061 (PMC12582370; doi:10.1093/gpbjnl/qzaf061)
Supplement: qzaf061_Supplementary_Data [file qzaf061_supplementary_data.zip › Fig S13.pdf]

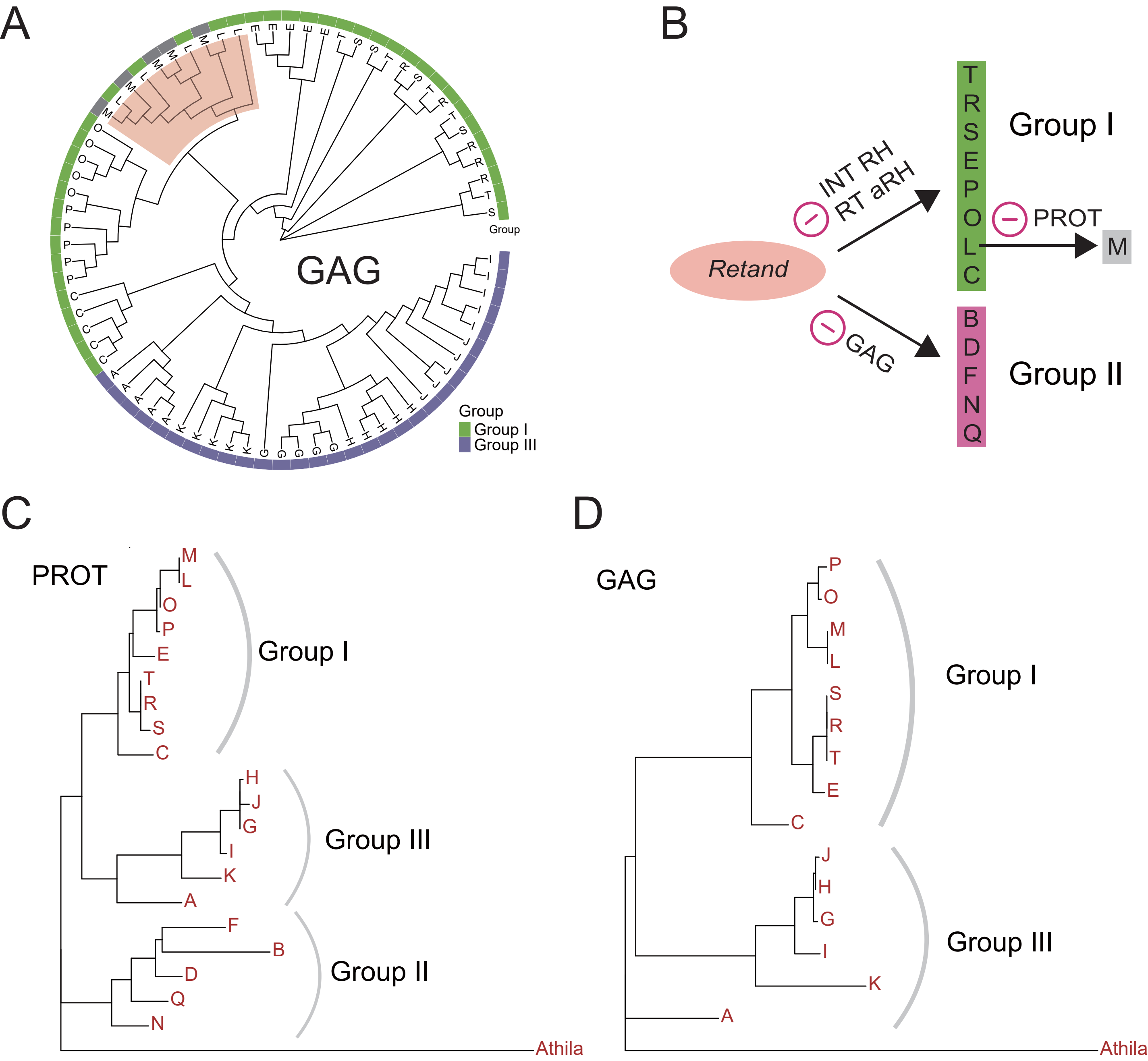
A
GAG
Group
Group
Group I
Group III
B
Retand
INT RH
RT aRH
GAG
PROT
T R S E P O L C
Group I
M
B D F N Q
Group II
C
PROT
M
L
O
P
E
T
R
S
C
Group I
H
J
G
I
K
A
Group III
F
B
D
Q
N
Group II
Athila
D
GAG
P
O
M
L
S
R
T
E
C
Group I
J
H
G
I
K
Group III
A
Athila
