# Supplementary material for: Deciphering Complex Interactions Between LTR Retrotransposons and Three Papaver Species Using LTR_Stream
Source: Genomics Proteomics Bioinformatics. 2025 Jul 8;23(4):qzaf061. doi: 10.1093/gpbjnl/qzaf061 (PMC12582370; doi:10.1093/gpbjnl/qzaf061)
Supplement: qzaf061_Supplementary_Data [file qzaf061_supplementary_data.zip › Fig S17.pdf]

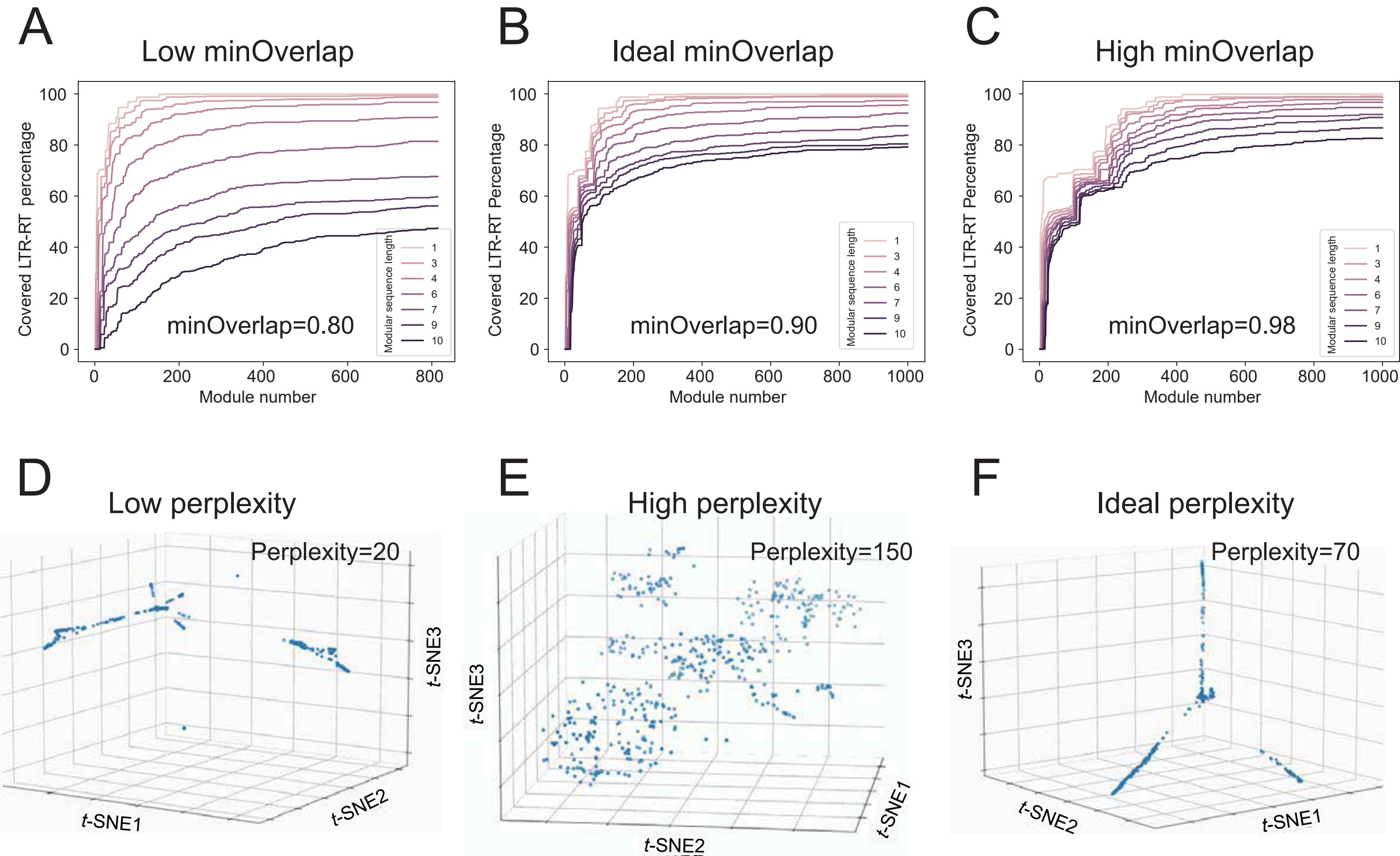
A
Low minOverlap
Covered LTR-RT percentage
Module number
minOverlap=0.80
Modular sequence length
1
3
4
6
7
9
10
B
Ideal minOverlap
Covered LTR-RT Percentage
Module number
minOverlap=0.90
C
High minOverlap
Covered LTR-RT Percentage
Module number
minOverlap=0.98
D
Low perplexity
Perplexity=20
t-SNE1
t-SNE2
t-SNE3
E
High perplexity
Perplexity=150
F
Ideal perplexity
Perplexity=70
