# Supplementary material for: Deciphering Complex Interactions Between LTR Retrotransposons and Three Papaver Species Using LTR_Stream
Source: Genomics Proteomics Bioinformatics. 2025 Jul 8;23(4):qzaf061. doi: 10.1093/gpbjnl/qzaf061 (PMC12582370; doi:10.1093/gpbjnl/qzaf061)
Supplement: qzaf061_Supplementary_Data [file qzaf061_supplementary_data.zip › File S1.pdf]

Module b1062676 of SG1 *P. so* TCP and bZIP motif enrichment

Motif ID	TF type	Motif	*P* value	Target%	Background%
MP00636	TCP	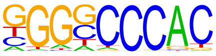	1E–138	72.06%	34.47%
MP00655	TCP	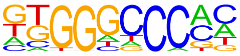	1E–96	75.22%	44.27%
MP00063	TCP	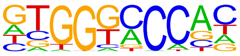	1E–61	97.72%	83.23%
MP00303	bZIP	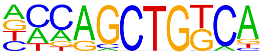	1E–59	77.27%	53.63%
MP00665	bZIP	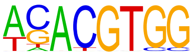	1E–56	70.42%	46.77%
MP00040	bZIP	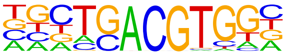	1E–45	81.96%	62.47%
MP00129	bZIP	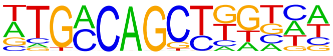	1E–29	78.38%	62.67%
MP00064	TCP	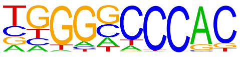	1E–27	86.12%	72.47%

Module b388131 of SG1 *P. so* TCP and bZIP motif enrichment

Motif ID	TF type	Motif	*P* value	Target%	Background%
MP00303	bZIP	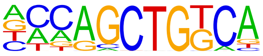	0E+00	78.84%	5.43%
MP00129	bZIP	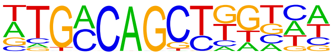	0E+00	79.21%	6.6%
MP00039	bZIP	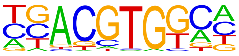	0E+00	91.63%	24.77%
MP00037	bZIP	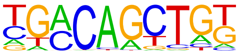	1E–323	85.19%	20.03%

Module b761785 of SG1 *P. so* TCP and bZIP motif enrichment

Motif ID	TF type	Motif	*P* value	Target%	Background%
MP00586	bZIP	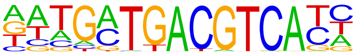	1E–265	71.8%	10.6%
MP00040	bZIP	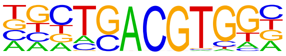	1E–162	86.13%	35.13%
MP00019	bZIP	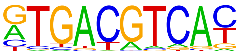	1E–86	74.5%	36.53%
MP00063	TCP	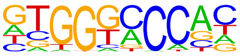	1E–25	72.74%	52.83%

Module b759926 of SG1 *P. so* TCP and bZIP motif enrichment

Motif ID	TF type	Motif	*P* value	Target%	Background%
MP00040	bZIP	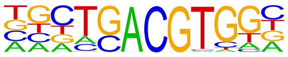	1E–182	83.71%	54.47%
MP00039	bZIP	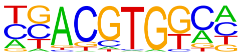	1E–40	99.04%	94%

Module b936193 of SG1 *P. so* TCP and bZIP motif enrichment

Motif ID	TF type	Motif	*P* value	Target%	Background%
MP00039	bZIP	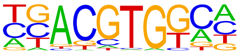	1E–43	82.09%	46.2%

Module b144725 of SG1 *P. so* TCP and bZIP motif enrichment

Motif ID	TF type	Motif	*P* value	Target%	Background%
MP00039	bZIP	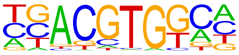	0E+00	95.46%	55.9%
MP00037	bZIP	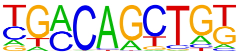	1E–128	75.34%	50.33%

Module b765177 of SG1 *P. so* TCP and bZIP motif enrichment

Motif ID	TF type	Motif	*P* value	Target%	Background%
MP00345	bZIP	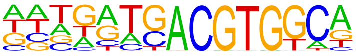	0E+00	88.58%	1.8%
MP00409	bZIP	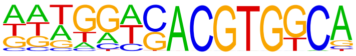	0E+00	86.84%	0.73%
MP00186	bZIP	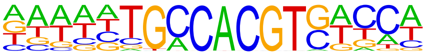	0E+00	87.2%	1.1%
MP00040	bZIP	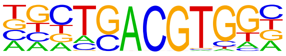	0E+00	93.41%	6.8%
MP00294	bZIP	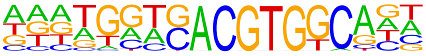	0E+00	86.31%	0.83%
MP00173	bZIP	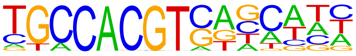	0E+00	87.12%	1.7%
MP00647	bZIP	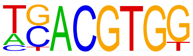	0E+00	88.11%	3.43%
MP00039	bZIP	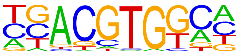	0E+00	98.22%	20.8%

Module b365303 of SG1 *P. so* TCP and bZIP motif enrichment

No TCP or bZIP related motif that show *P* value smaller than 1E–10 and more than 70% percentage in module sequences.

Module b1258177 of SG1 *P. so* TCP and bZIP motif enrichment

Motif ID	TF type	Motif	*P* value	Target%	Background%
MP00419	bZIP	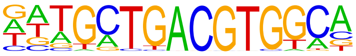	1E–176	71.86%	2.63%
MP00638	TCP	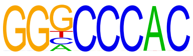	1E–168	83.27%	7.23%
MP00239	bZIP	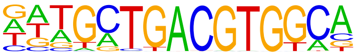	1E–165	71.48%	3.33%
MP00524	TCP	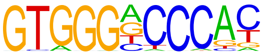	1E–158	78.71%	6.43%
MP00470	bZIP	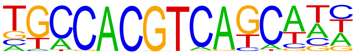	1E–156	71.86%	4.17%
MP00499	TCP	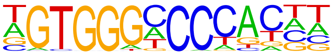	1E–155	81.75%	8.07%
MP00643	TCP	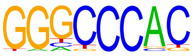	1E–154	85.93%	10.37%
MP00228	TCP	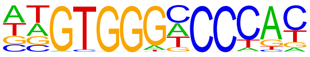	1E–147	82.89%	9.7%
MP00636	TCP	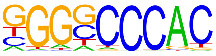	1E–142	88.21%	13.63%
MP00318	bZIP	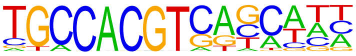	1E–142	70.34%	4.93%
MP00384	TCP	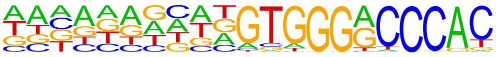	1E–137	79.85%	9.57%
MP00224	TCP	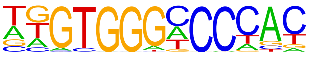	1E–137	82.89%	11.27%
MP00117	bZIP	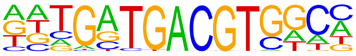	1E–128	71.86%	7.07%
MP00641	TCP	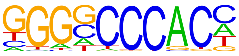	1E–128	87.07%	15.57%
MP00345	bZIP	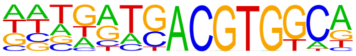	1E–127	76.05%	9.07%
MP00491	bZIP	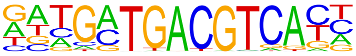	1E–117	71.48%	8.47%
MP00655	TCP	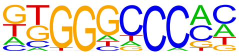	1E–111	86.31%	18.5%
MP00502	bZIP	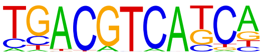	1E–111	85.55%	18.03%

Motif ID	TF type	Motif	P value	Target%	Background%
MP00635	TCP	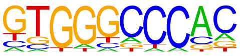	1E–110	80.61%	14.7%
MP00647	bZIP	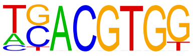	1E–90	77.57%	17.03%
MP00040	bZIP	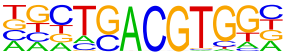	1E–85	90.11%	29.53%
MP00064	TCP	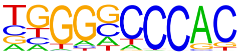	1E–80	93.92%	36.03%
MP00665	bZIP	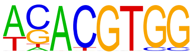	1E–77	77.19%	20.3%
MP00019	bZIP	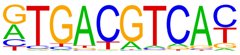	1E–68	84.03%	29.33%
MP00063	TCP	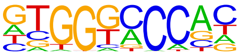	1E–58	93.92%	45.6%
MP00039	bZIP	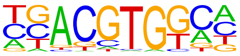	1E–31	99.24%	72.1%
MP00037	bZIP	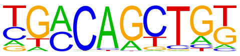	1E–21	90.87%	63.8%

Module b454270 of SG1 *P. so* TCP and bZIP motif enrichment

Motif ID	TF type	Motif	*P* value	Target%	Background%
MP00063	TCP	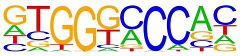	1E–117	91.59%	54.5%

Module b213833 of SG1 *P. so* TCP and bZIP motif enrichment

Motif ID	TF type	Motif	*P* value	Target%	Background%
MP00039	bZIP	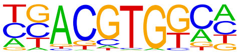	1E–29	78.44%	51.77%

Module b956 of SG1 *P. so* TCP and bZIP motif enrichment

No TCP or bZIP related motif that show *P* value smaller than 1E–10 and more than 70% percentage in module sequences.

Module b1228119 of SG1 *P. so* TCP and bZIP motif enrichment

Motif ID	TF type	Motif	*P* value	Target%	Background%
MP00215	TCP	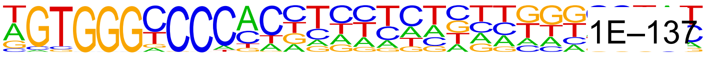	1E–137	72.46%	4.93%
MP00316	TCP	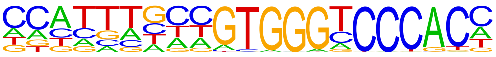	1E–136	70.34%	4.3%
MP00639	TCP	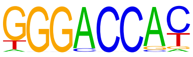	1E–128	98.31%	23.07%
MP00384	TCP	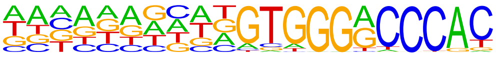	1E–119	85.59%	13.8%
MP00655	TCP	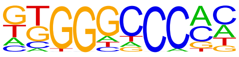	1E–114	97.88%	26.53%
MP00638	TCP	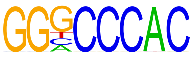	1E–112	76.69%	10.03%
MP00499	TCP	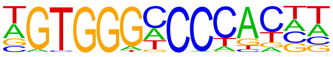	1E–110	80.51%	12.43%
MP00524	TCP	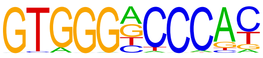	1E–110	75.85%	9.93%
MP00228	TCP	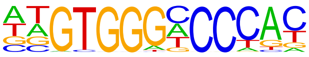	1E–109	82.63%	14.07%
MP00636	TCP	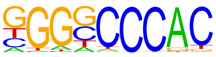	1E–101	88.14%	20.1%
MP00643	TCP	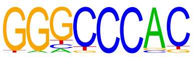	1E–99	80.51%	14.83%
MP00224	TCP	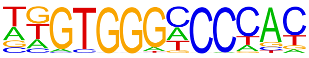	1E–94	82.2%	17.13%
MP00635	TCP	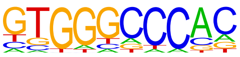	1E–84	83.9%	21.2%
MP00641	TCP	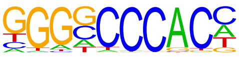	1E–79	84.75%	23.6%
MP00064	TCP	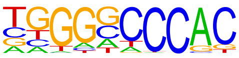	1E–63	99.15%	49.93%
MP00665	bZIP	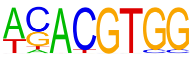	1E–61	83.9%	29.6%
MP00647	bZIP	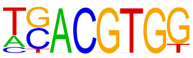	1E–55	80.08%	28.13%
MP00184	bZIP	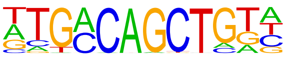	1E–53	73.73%	23.7%

Motif ID	TF type	Motif	P value	Target%	Background%
MP00063	TCP	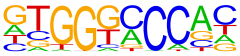	1E–50	100%	59.67%
MP00129	bZIP	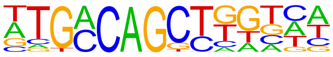	1E–47	88.14%	41.07%
MP00040	bZIP	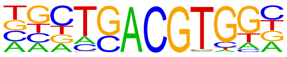	1E–39	85.17%	41.63%
MP00303	bZIP	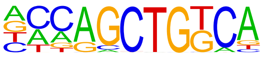	1E–39	77.12%	33%
MP00037	bZIP	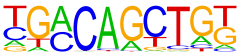	1E–16	98.73%	80.47%

Module b177423 of SG1 *P. so* TCP and bZIP motif enrichment

No TCP or bZIP related motif that show *P* value smaller than 1E–10 and more than 70% percentage in module sequences.

Module b1192663 of SG1 *P. so* TCP and bZIP motif enrichment

Motif ID	TF type	Motif	*P* value	Target%	Background%
MP00524	TCP	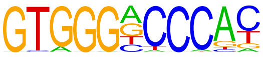	1E–80	75.89%	17.7%
MP00641	TCP	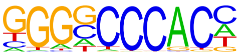	1E–64	91.3%	38.47%
MP00636	TCP	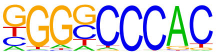	1E–53	82.21%	32.87%
MP00639	TCP	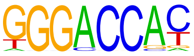	1E–37	78.66%	37.23%
MP00064	TCP	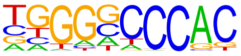	1E–14	91.7%	70.47%
MP00063	TCP	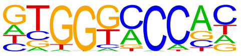	1E–12	97.63%	82.67%

Module b1062676 of SG2 *P. so* TCP and bZIP motif enrichment

Motif ID	TF type	Motif	*P* value	Target%	Background%
MP00643	TCP	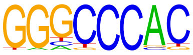	1E–206	70.53%	23.53%
MP00636	TCP	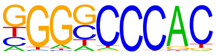	1E–161	74.2%	32.2%
MP00639	TCP	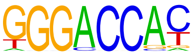	1E–106	70.92%	36.67%
MP00655	TCP	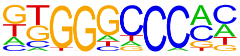	1E–98	76.23%	43.87%
MP00040	bZIP	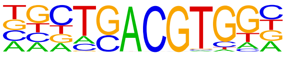	1E–66	83.76%	59.1%
MP00063	TCP	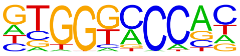	1E–64	98.36%	83.33%
MP00303	bZIP	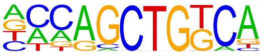	1E–63	78.06%	52.87%
MP00665	bZIP	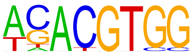	1E–52	70.92%	47.3%
MP00129	bZIP	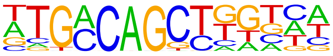	1E–26	79.31%	63.87%
MP00064	TCP	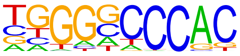	1E–18	85.33%	73.93%

Module b388131 of SG2 *P. so* TCP and bZIP motif enrichment

Motif ID	TF type	Motif	*P* value	Target%	Background%
MP00303	bZIP	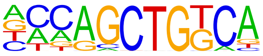	0E+00	80.08%	4.1%
MP00129	bZIP	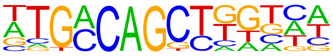	0E+00	80.38%	6.37%
MP00037	bZIP	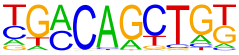	0E+00	88.21%	17.9%
MP00039	bZIP	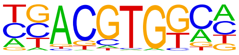	0E+00	91.28%	22.57%

Module b761785 of SG2 *P. so* TCP and bZIP motif enrichment

Motif ID	TF type	Motif	*P* value	Target%	Background%
MP00040	bZIP	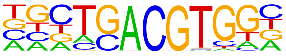	1E–157	85.44%	30.7%
MP00019	bZIP	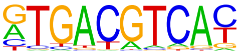	1E–79	72.2%	32.8%

Module b936193 of SG2 *P. so* TCP and bZIP motif enrichment

Motif ID	TF type	Motif	*P* value	Target%	Background%
MP00039	bZIP	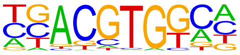	1E–49	83.76%	47.1%

Module b365303 of SG2 *P. so* TCP and bZIP motif enrichment

No TCP or bZIP related motif that show *P* value smaller than 1E–10 and more than 70% percentage in module sequences.

Module b213833 of SG2 *P. so* TCP and bZIP motif enrichment

Motif ID	TF type	Motif	*P* value	Target%	Background%
MP00039	bZIP	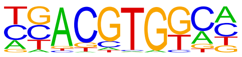	1E–18	76.64%	52.1%

Module b144725 of SG2 *P. so* TCP and bZIP motif enrichment

No TCP or bZIP related motif that show *P* value smaller than 1E–10 and more than 70% percentage in module sequences.

Module b1390031 of SG3 *P. se* TCP and bZIP motif enrichment

Motif ID	TF type	Motif	*P* value	Target%	Background%
MP00635	TCP	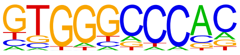	1E–105	89.18%	26.43%
MP00384	TCP	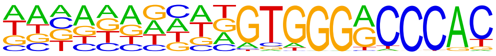	1E–89	76.07%	19.27%
MP00655	TCP	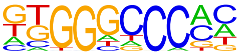	1E–88	91.8%	34.7%
MP00345	bZIP	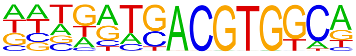	1E–83	75.08%	20.07%
MP00647	bZIP	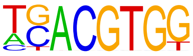	1E–80	89.51%	34.63%
MP00184	bZIP	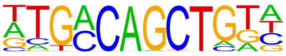	1E–70	81.97%	29.9%
MP00665	bZIP	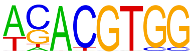	1E–69	90.49%	39.83%
MP00040	bZIP	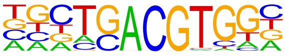	1E–69	96.39%	49%
MP00019	bZIP	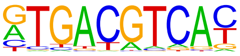	1E–43	94.75%	58.77%
MP00303	bZIP	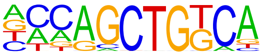	1E–40	81.31%	41.97%
MP00064	TCP	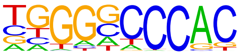	1E–30	93.77%	64.53%
MP00063	TCP	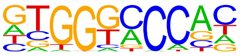	1E–22	96.72%	75.43%
MP00129	bZIP	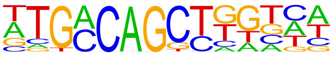	1E–10	73.11%	53.97%

Module b1388073 of SG3 *P. se* TCP and bZIP motif enrichment

Motif ID	TF type	Motif	*P* value	Target%	Background%
MP00493	bZIP	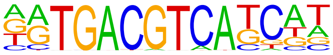	1E−162	92.02%	10.77%
MP00492	bZIP	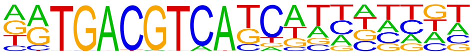	1E−148	92.02%	12.97%
MP00157	bZIP	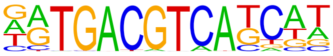	1E−143	92.02%	13.87%
MP00349	bZIP	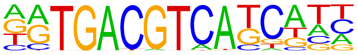	1E−143	92.02%	13.87%
MP00247	bZIP	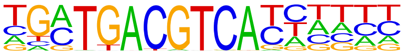	1E−127	92.86%	17.93%
MP00131	bZIP	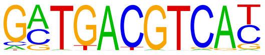	1E−121	92.86%	19.23%
MP00491	bZIP	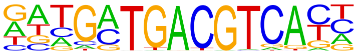	1E−112	90.34%	19.37%
MP00586	bZIP	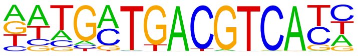	1E−104	92.44%	23.5%
MP00228	TCP	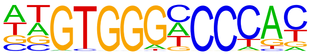	1E−84	82.35%	20.23%
MP00224	TCP	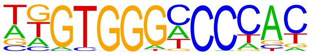	1E−77	84.03%	23.67%
MP00639	TCP	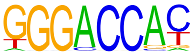	1E−68	91.18%	34.5%
MP00635	TCP	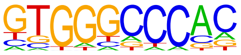	1E−61	83.61%	29.47%
MP00655	TCP	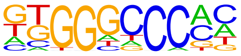	1E−60	91.18%	38.3%
MP00502	bZIP	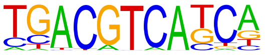	1E−57	91.6%	40.5%
MP00040	bZIP	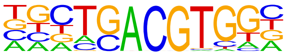	1E−41	94.96%	54.63%
MP00019	bZIP	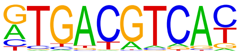	1E−29	95.8%	64.43%
MP00063	TCP	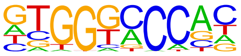	1E−21	99.58%	79.03%
MP00303	bZIP	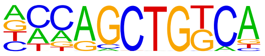	1E−13	71.43%	46.37%

Module b1062676 of SG3 *P. se* TCP and bZIP motif enrichment

Motif ID	TF type	Motif	*P* value	Target%	Background%
MP00636	TCP	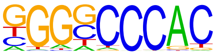	1E–54	81.12%	30.87%
MP00655	TCP	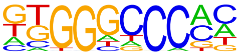	1E–35	83.53%	43.37%
MP00665	bZIP	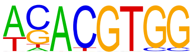	1E–16	75.9%	49.1%
MP00040	bZIP	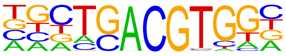	1E–14	85.14%	61.3%
MP00502	bZIP	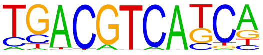	1E–12	70.28%	46.13%

Module b388131 of SG3 *P. se* TCP and bZIP motif enrichment

Motif ID	TF type	Motif	*P* value	Target%	Background%
MP00039	bZIP	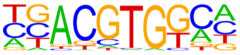	1E–56	78.57%	25.23%

Module b1390031 of SG4 *P. se* TCP and bZIP motif enrichment

Motif ID	TF type	Motif	*P* value	Target%	Background%
MP00635	TCP	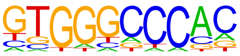	1E–88	85.17%	25.97%
MP00647	bZIP	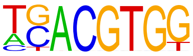	1E–85	91.38%	33.6%
MP00345	bZIP	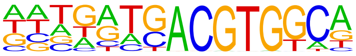	1E–75	73.1%	19.8%
MP00040	bZIP	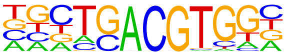	1E–73	97.59%	47.73%
MP00184	bZIP	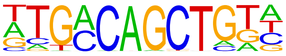	1E–70	83.79%	30.67%
MP00655	TCP	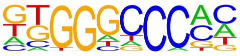	1E–69	88.28%	35.9%
MP00665	bZIP	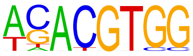	1E–65	89.66%	39.1%
MP00303	bZIP	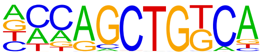	1E–48	84.14%	40.07%
MP00019	bZIP	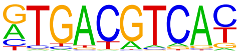	1E–37	91.72%	56.07%
MP00064	TCP	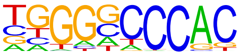	1E–21	90%	63.97%
MP00063	TCP	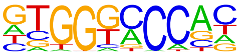	1E–18	95.86%	76.27%
MP00129	bZIP	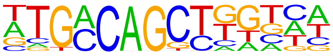	1E–16	77.93%	52.7%

Module b1388073 of SG4 *P. se* TCP and bZIP motif enrichment

Motif ID	TF type	Motif	*P* value	Target%	Background%
MP00493	bZIP	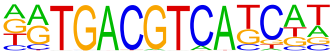	1E–150	92.72%	9.87%
MP00349	bZIP	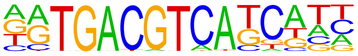	1E–138	92.72%	11.9%
MP00492	bZIP	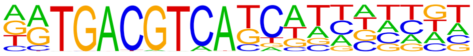	1E–138	92.72%	11.9%
MP00157	bZIP	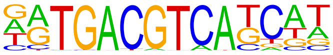	1E–137	92.72%	12.2%
MP00247	bZIP	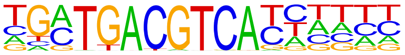	1E–120	93.2%	16.1%
MP00131	bZIP	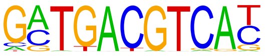	1E–113	92.72%	17.23%
MP00491	bZIP	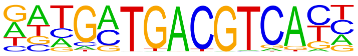	1E–107	91.26%	17.67%
MP00586	bZIP	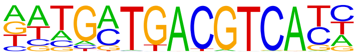	1E–103	92.72%	20.1%
MP00228	TCP	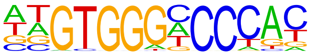	1E–67	79.13%	20.07%
MP00224	TCP	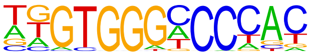	1E–66	83.01%	23.5%
MP00502	bZIP	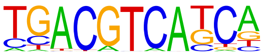	1E–52	91.26%	38.5%
MP00639	TCP	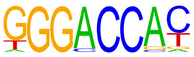	1E–49	87.86%	35.97%
MP00635	TCP	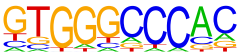	1E–44	80.1%	30.8%
MP00655	TCP	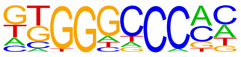	1E–43	88.83%	40.6%
MP00040	bZIP	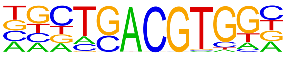	1E–34	94.66%	55.1%
MP00019	bZIP	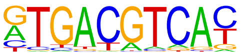	1E–25	94.17%	61.33%
MP00063	TCP	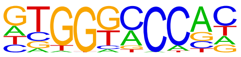	1E–17	99.51%	80.5%
MP00303	bZIP	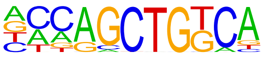	1E–13	74.27%	47.47%

Module b1062676 of SG4 *P. se* TCP and bZIP motif enrichment

Motif ID	TF type	Motif	*P* value	Target%	Background%
MP00636	TCP	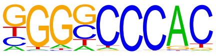	1E–57	78.02%	28.43%
MP00655	TCP	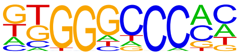	1E–31	79.49%	42.87%
MP00665	bZIP	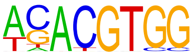	1E–17	75.46%	48.2%
MP00647	bZIP	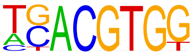	1E–17	70.7%	43.4%
MP00040	bZIP	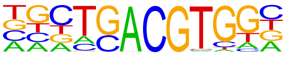	1E–16	83.15%	58.77%
MP00063	TCP	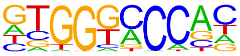	1E–13	97.8%	83.53%

Module b388131 of SG4 *P. se* TCP and bZIP motif enrichment

Motif ID	TF type	Motif	*P* value	Target%	Background%
MP00303	bZIP	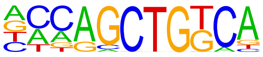	1E–136	73.87%	4.73%
MP00129	bZIP	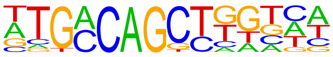	1E–126	75.23%	6.4%
MP00037	bZIP	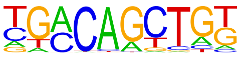	1E–77	77.48%	17.17%
MP00039	bZIP	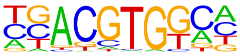	1E–73	83.33%	23.07%
