# Supplementary material for: Deciphering Complex Interactions Between LTR Retrotransposons and Three Papaver Species Using LTR_Stream
Source: Genomics Proteomics Bioinformatics. 2025 Jul 8;23(4):qzaf061. doi: 10.1093/gpbjnl/qzaf061 (PMC12582370; doi:10.1093/gpbjnl/qzaf061)
Supplement: qzaf061_Supplementary_Data [file qzaf061_supplementary_data.zip › File S2.pdf]

TAD-like boundary of SG1 *P. so* TCP and bZIP motif enrichment

Motif ID	TF type	Motif	*P* value	Target%	Background%
MP00586	bZIP	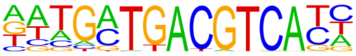	0E+00	90.08%	49.16%
MP00173	bZIP	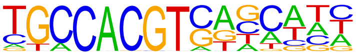	0E+00	90.21%	49.44%
MP00491	bZIP	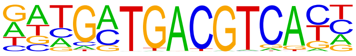	0E+00	87.71%	45.38%
MP00247	bZIP	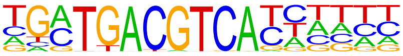	0E+00	86.32%	43.18%
MP00131	bZIP	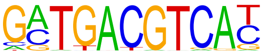	0E+00	86.72%	43.96%
MP00345	bZIP	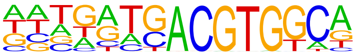	0E+00	90.86%	51.66%
MP00169	TCP	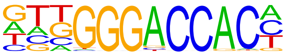	0E+00	89.65%	49.96%
MP00349	bZIP	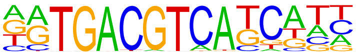	0E+00	76.2%	31.04%
MP00203	TCP	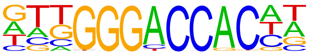	0E+00	83.26%	40.52%
MP00512	bZIP	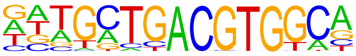	0E+00	74.39%	29.38%
MP00157	bZIP	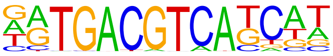	0E+00	78.41%	34.28%
MP00117	bZIP	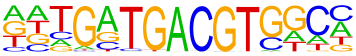	0E+00	84.29%	42.3%
MP00318	bZIP	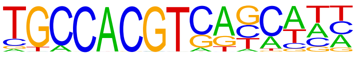	0E+00	74.8%	30.1%
MP00384	TCP	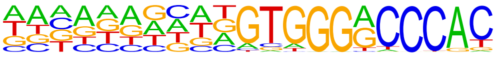	0E+00	88.89%	49.64%
MP00126	bZIP	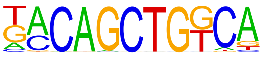	0E+00	77.32%	33.2%
MP00186	bZIP	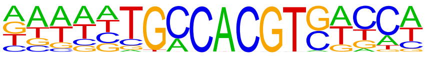	0E+00	78.77%	35.02%
MP00492	bZIP	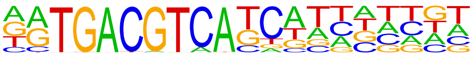	0E+00	75.16%	30.82%
MP00267	bZIP	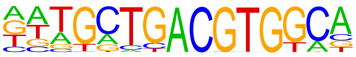	0E+00	73.53%	29.1%

Motif ID	TF type	Motif	*P* value	Target%	Background%
MP00062	TCP	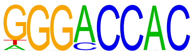	0E+00	76.97%	33.24%
MP00224	TCP	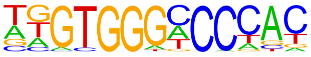	0E+00	92.53%	56.96%
MP00228	TCP	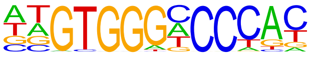	0E+00	88.76%	50.34%
MP00524	TCP	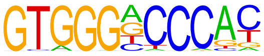	0E+00	81.09%	38.94%
MP00499	TCP	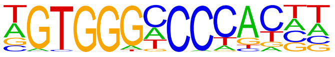	0E+00	85.9%	45.82%
MP00643	TCP	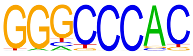	0E+00	88.01%	49.54%
MP00636	TCP	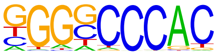	0E+00	94.48%	62.2%
MP00635	TCP	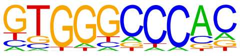	0E+00	95.54%	64.82%
MP00470	bZIP	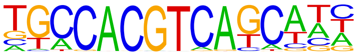	0E+00	72.48%	29.5%
MP00006	TCP	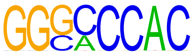	0E+00	75.3%	32.88%
MP00390	TCP	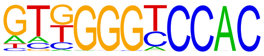	0E+00	72.22%	29.56%
MP00639	TCP	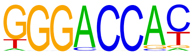	0E+00	96.34%	67.18%
MP00497	TCP	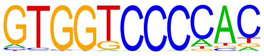	0E+00	74.09%	31.88%
MP00184	bZIP	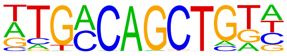	0E+00	97.44%	70.7%
MP00641	TCP	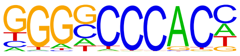	0E+00	96.74%	69.7%
MP00638	TCP	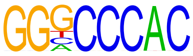	0E+00	76.15%	36.1%
MP00614	TCP	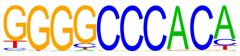	0E+00	75.29%	35.28%
MP00502	bZIP	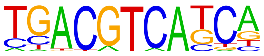	0E+00	98.35%	75.54%
MP00665	bZIP	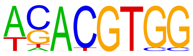	0E+00	98.51%	76.18%

Motif ID	TF type	Motif	*P* value	Target%	Background%
MP00647	bZIP	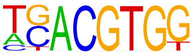	0E+00	97.94%	75.08%
MP00655	TCP	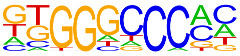	0E+00	97.35%	73.36%
MP00303	bZIP	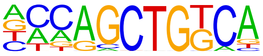	0E+00	99.33%	84.46%
MP00040	bZIP	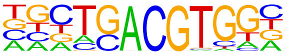	1E–281	99.63%	89.42%
MP00129	bZIP	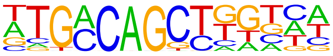	1E–240	99.83%	91.68%
MP00019	bZIP	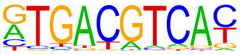	1E–214	99.83%	92.48%
MP00064	TCP	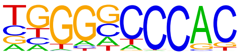	1E–129	99.88%	95.3%
MP00063	TCP	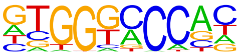	1E–51	99.94%	98.06%
MP00037	bZIP	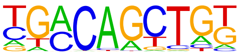	1E–14	99.97%	99.4%

TAD-like boundary of SG2 *P. so* TCP and bZIP motif enrichment

Motif ID	TF type	Motif	*P* value	Target%	Background%
MP00586	bZIP	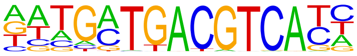	0E+00	90.08%	46.92%
MP00491	bZIP	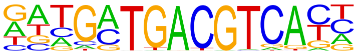	0E+00	87.82%	43.26%
MP00131	bZIP	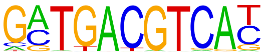	0E+00	86.75%	42.2%
MP00247	bZIP	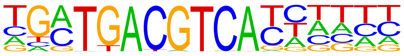	0E+00	85.9%	41.38%
MP00349	bZIP	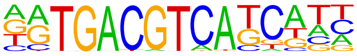	0E+00	78.65%	31.72%
MP00173	bZIP	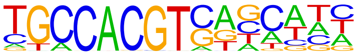	0E+00	89.9%	48.3%
MP00117	bZIP	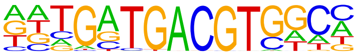	0E+00	83.14%	38%
MP00499	TCP	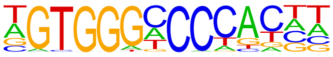	0E+00	86.02%	42.26%
MP00203	TCP	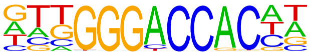	0E+00	82.1%	37.02%
MP00157	bZIP	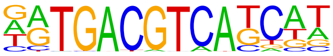	0E+00	78.69%	32.68%
MP00384	TCP	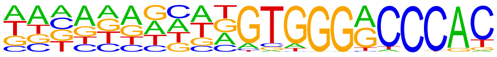	0E+00	90.78%	50.66%
MP00224	TCP	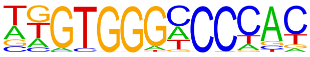	0E+00	92.5%	54%
MP00169	TCP	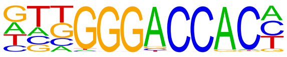	0E+00	89.83%	49.08%
MP00186	bZIP	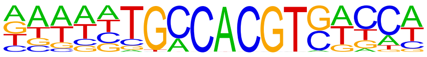	0E+00	78.58%	32.76%
MP00228	TCP	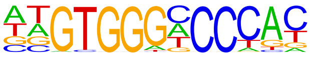	0E+00	88.85%	47.5%
MP00345	bZIP	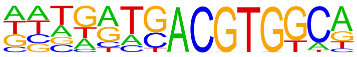	0E+00	91.38%	52.08%
MP00492	bZIP	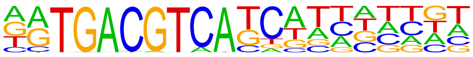	0E+00	76.87%	30.92%
MP00524	TCP	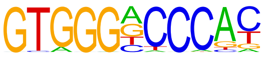	0E+00	83.4%	39.36%

Motif ID	TF type	Motif	*P* value	Target%	Background%
MP00643	TCP	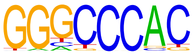	0E+00	88.86%	47.86%
MP00062	TCP	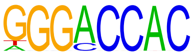	0E+00	78.78%	33.42%
MP00512	bZIP	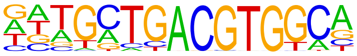	0E+00	74.59%	29.3%
MP00126	bZIP	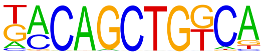	0E+00	80.3%	36.5%
MP00318	bZIP	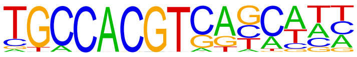	0E+00	74.67%	29.94%
MP00470	bZIP	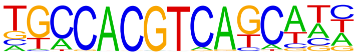	0E+00	70.45%	25.48%
MP00267	bZIP	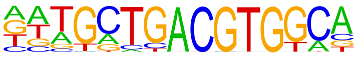	0E+00	73.4%	28.7%
MP00493	bZIP	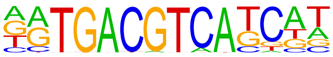	0E+00	71.76%	27.04%
MP00636	TCP	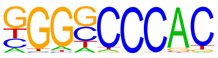	0E+00	94.96%	61.78%
MP00635	TCP	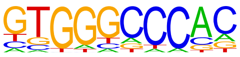	0E+00	95.41%	62.84%
MP00638	TCP	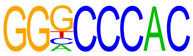	0E+00	74.76%	30.98%
MP00006	TCP	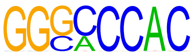	0E+00	72.8%	29.34%
MP00390	TCP	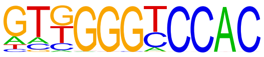	0E+00	74.25%	31.02%
MP00497	TCP	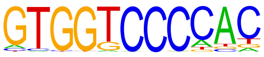	0E+00	73.46%	30.16%
MP00326	TCP	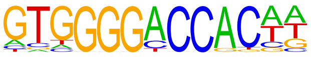	0E+00	70.45%	26.96%
MP00614	TCP	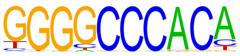	0E+00	73.22%	30.12%
MP00184	bZIP	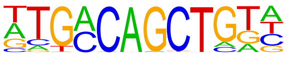	0E+00	96.98%	68.52%
MP00639	TCP	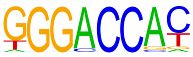	0E+00	96.35%	66.9%
MP00641	TCP	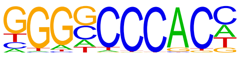	0E+00	97.46%	70.88%

Motif ID	TF type	Motif	*P* value	Target%	Background%
MP00502	bZIP	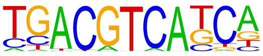	0E+00	98.26%	74.26%
MP00647	bZIP	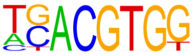	0E+00	98.02%	74.4%
MP00655	TCP	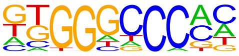	0E+00	97.77%	73.74%
MP00665	bZIP	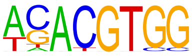	0E+00	98.56%	78.76%
MP00303	bZIP	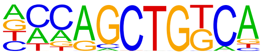	0E+00	99.14%	81.86%
MP00040	bZIP	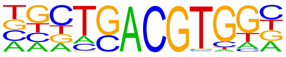	1E–283	99.51%	88.14%
MP00129	bZIP	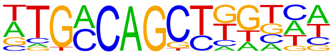	1E–251	99.7%	90.1%
MP00019	bZIP	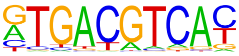	1E–215	99.68%	91.18%
MP00064	TCP	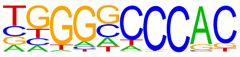	1E–105	99.84%	95.62%
MP00063	TCP	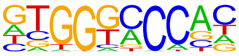	1E–49	99.87%	97.72%
MP00037	bZIP	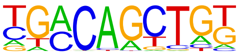	1E–10	99.96%	99.48%

TAD-like boundary of SG3 *P. se* TCP and bZIP motif enrichment

Motif ID	TF type	Motif	*P* value	Target%	Background%
MP00586	bZIP	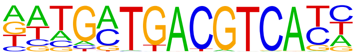	0E+00	90.92%	48.38%
MP00491	bZIP	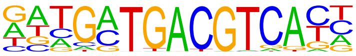	0E+00	87.84%	43.44%
MP00499	TCP	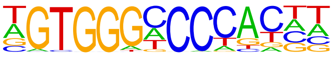	0E+00	85%	39.46%
MP00117	bZIP	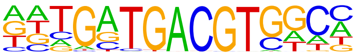	0E+00	82.75%	36.4%
MP00131	bZIP	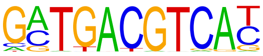	0E+00	86.67%	42.2%
MP00384	TCP	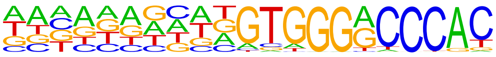	0E+00	91.23%	49.84%
MP00228	TCP	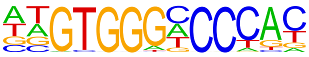	0E+00	88.58%	45.38%
MP00173	bZIP	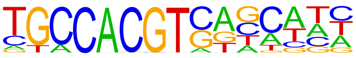	0E+00	88.82%	45.8%
MP00345	bZIP	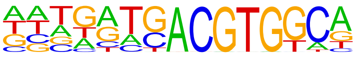	0E+00	90.65%	49.1%
MP00247	bZIP	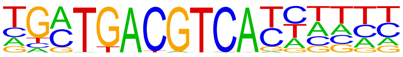	0E+00	86%	41.72%
MP00224	TCP	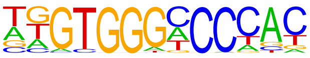	0E+00	91.52%	50.98%
MP00643	TCP	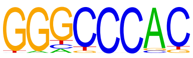	0E+00	85.21%	40.88%
MP00169	TCP	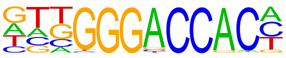	0E+00	87.99%	45.12%
MP00492	bZIP	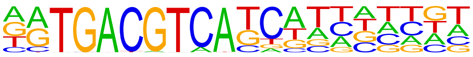	0E+00	77.47%	31.14%
MP00524	TCP	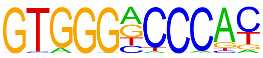	0E+00	79.9%	34.24%
MP00203	TCP	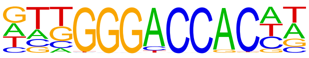	0E+00	80.59%	35.16%
MP00349	bZIP	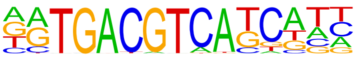	0E+00	78.09%	32.14%
MP00318	bZIP	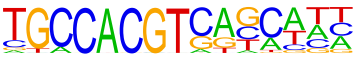	0E+00	73.21%	26.76%

Motif ID	TF type	Motif	*P* value	Target%	Background%
MP00157	bZIP	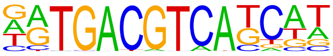	0E+00	78.66%	33.02%
MP00512	bZIP	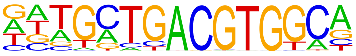	0E+00	74.13%	28%
MP00267	bZIP	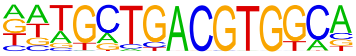	0E+00	72.85%	26.72%
MP00186	bZIP	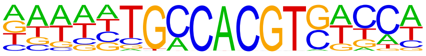	0E+00	75.48%	29.84%
MP00636	TCP	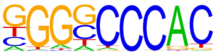	0E+00	93.58%	57.08%
MP00326	TCP	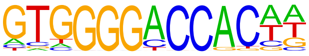	0E+00	70.17%	24.68%
MP00635	TCP	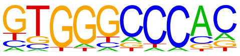	0E+00	94.79%	60.02%
MP00062	TCP	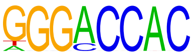	0E+00	75.86%	31.2%
MP00470	bZIP	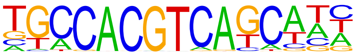	0E+00	70.18%	25.2%
MP00126	bZIP	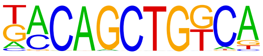	0E+00	75.79%	31.46%
MP00497	TCP	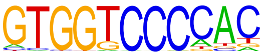	0E+00	72.76%	28.14%
MP00493	bZIP	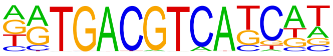	0E+00	71.49%	26.9%
MP00390	TCP	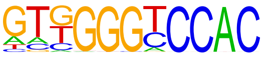	0E+00	71.41%	26.84%
MP00184	bZIP	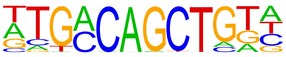	0E+00	96.86%	66.56%
MP00639	TCP	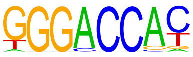	0E+00	96.64%	67.3%
MP00502	bZIP	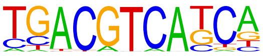	0E+00	98.21%	72.14%
MP00641	TCP	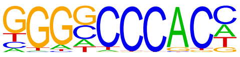	0E+00	96.88%	69.24%
MP00655	TCP	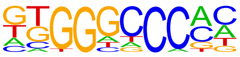	0E+00	97.77%	71.84%
MP00647	bZIP	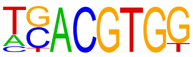	0E+00	97.88%	72.62%

Motif ID	TF type	Motif	P value	Target%	Background%
MP00665	bZIP	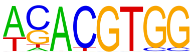	0E+00	98.72%	77.36%
MP00303	bZIP	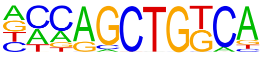	0E+00	99.14%	80.16%
MP00040	bZIP	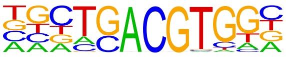	1E–320	99.54%	86.08%
MP00129	bZIP	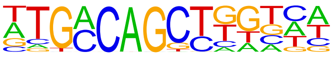	1E–225	99.75%	90.44%
MP00019	bZIP	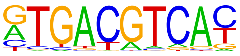	1E–199	99.78%	91.5%
MP00064	TCP	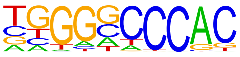	1E–98	99.88%	95.7%
MP00063	TCP	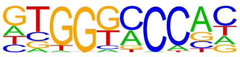	1E–41	99.98%	98.36%

TAD-like boundary of SG4 *P. se* TCP and bZIP motif enrichment

Motif ID	TF type	Motif	*P* value	Target%	Background%
MP00491	bZIP	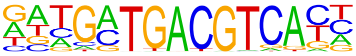	0E+00	86.76%	41.1%
MP00247	bZIP	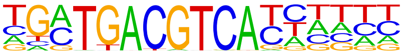	0E+00	85.78%	40.1%
MP00169	TCP	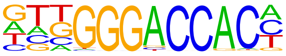	0E+00	89.16%	45.42%
MP00643	TCP	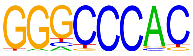	0E+00	85.2%	39.7%
MP00586	bZIP	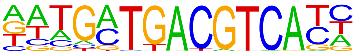	0E+00	90.54%	48.26%
MP00203	TCP	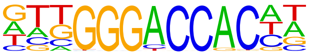	0E+00	81.32%	34.6%
MP00131	bZIP	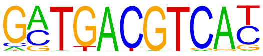	0E+00	86.01%	41.08%
MP00173	bZIP	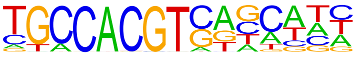	0E+00	89.72%	47.12%
MP00224	TCP	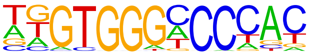	0E+00	91.91%	51.18%
MP00228	TCP	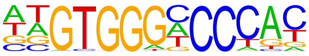	0E+00	88.87%	46.16%
MP00117	bZIP	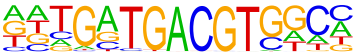	0E+00	81.79%	36%
MP00345	bZIP	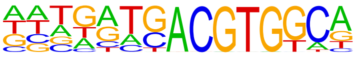	0E+00	91.31%	50.6%
MP00384	TCP	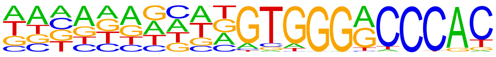	0E+00	91.41%	50.86%
MP00349	bZIP	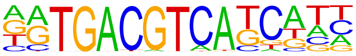	0E+00	77.83%	31.3%
MP00157	bZIP	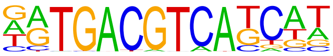	0E+00	78.14%	31.68%
MP00499	TCP	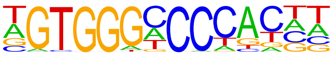	0E+00	85.17%	40.9%
MP00186	bZIP	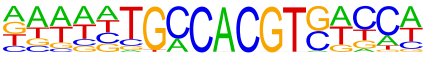	0E+00	78.04%	31.66%
MP00492	bZIP	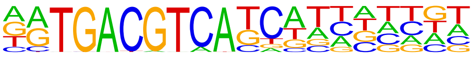	0E+00	77.19%	31.08%

Motif ID	TF type	Motif	*P* value	Target%	Background%
MP00524	TCP	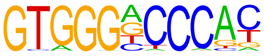	0E+00	79.85%	34.38%
MP00062	TCP	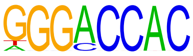	0E+00	77.63%	31.8%
MP00318	bZIP	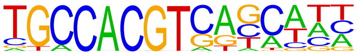	0E+00	74.94%	28.76%
MP00512	bZIP	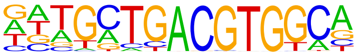	0E+00	75.66%	29.62%
MP00126	bZIP	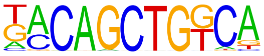	0E+00	77.3%	31.64%
MP00390	TCP	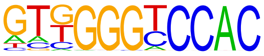	0E+00	72.85%	26.78%
MP00636	TCP	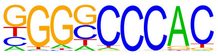	0E+00	92.89%	55.44%
MP00470	bZIP	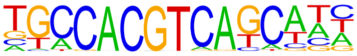	0E+00	70.86%	25.34%
MP00493	bZIP	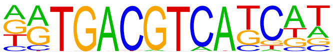	0E+00	71.09%	25.6%
MP00267	bZIP	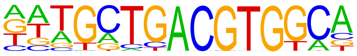	0E+00	73.75%	28.68%
MP00497	TCP	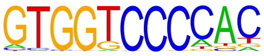	0E+00	74.41%	29.6%
MP00326	TCP	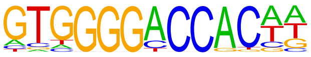	0E+00	71.65%	26.84%
MP00635	TCP	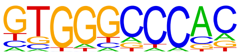	0E+00	95.04%	61%
MP00184	bZIP	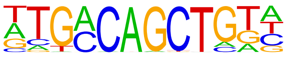	0E+00	96.93%	66.58%
MP00639	TCP	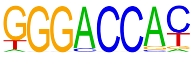	0E+00	97.2%	68.18%
MP00641	TCP	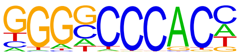	0E+00	96.7%	67.22%
MP00502	bZIP	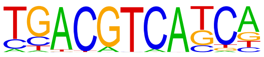	0E+00	98.12%	71.8%
MP00655	TCP	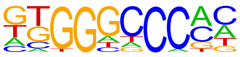	0E+00	97.94%	72.06%
MP00647	bZIP	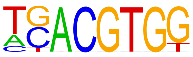	0E+00	98.09%	74.9%

Motif ID	TF type	Motif	*P* value	Target%	Background%
MP00665	bZIP	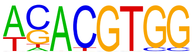	0E+00	98.85%	78.7%
MP00303	bZIP	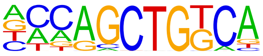	0E+00	99.02%	80.38%
MP00040	bZIP	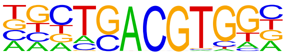	1E–286	99.65%	87.3%
MP00129	bZIP	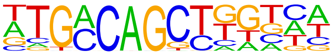	1E–226	99.7%	89.76%
MP00019	bZIP	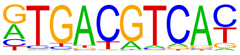	1E–185	99.77%	91.68%
MP00064	TCP	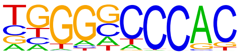	1E–83	99.77%	95.68%
MP00063	TCP	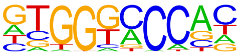	1E–39	99.98%	98.38%
