# Supplementary material for: Deciphering Complex Interactions Between LTR Retrotransposons and Three Papaver Species Using LTR_Stream
Source: Genomics Proteomics Bioinformatics. 2025 Jul 8;23(4):qzaf061. doi: 10.1093/gpbjnl/qzaf061 (PMC12582370; doi:10.1093/gpbjnl/qzaf061)
Supplement: qzaf061_Supplementary_Data [file qzaf061_supplementary_data.zip › Table S1.docx]

**Table S1 Simulated datasets**

| **Simulated LTR-RT dataset ID** | **Ancestral LTR-lineage** | **Number of branches** | **Number of simulated LTR-RTs** |
| --- | --- | --- | --- |
| 1 | *Ale* | 2 | 1646 |
| 2 | *Ale* | 3 | 2460 |
| 3 | *Ale* | 4 | 3292 |
| 4 | *CRM* | 2 | 1631 |
| 5 | *CRM* | 3 | 2413 |
| 6 | *CRM* | 4 | 3221 |
| 7 | *Tork* | 2 | 1607 |
| 8 | *Tork* | 3 | 2382 |
| 9 | *Tork* | 4 | 3186 |

*Note*: LTR-RT, long terminal repeat retrotransposon; ID, identifier; LTR, long terminal repeat.
