# Supplementary material for: Deciphering Complex Interactions Between LTR Retrotransposons and Three Papaver Species Using LTR_Stream
Source: Genomics Proteomics Bioinformatics. 2025 Jul 8;23(4):qzaf061. doi: 10.1093/gpbjnl/qzaf061 (PMC12582370; doi:10.1093/gpbjnl/qzaf061)
Supplement: qzaf061_Supplementary_Data [file qzaf061_supplementary_data.zip › Table S2.docx]

**Table S2 Assemblies of closely related species used for testing LTR_Stream**

| **Group** | **Species** | **Download links** |
| --- | --- | --- |
| *Gossypium* | *Gossypium herbaceum* | https://www.cottongen.org/cottongen_downloads/Gossypium_herbaceum/A1_WHU/assembly/Gherbaceum_A1-0076_WHUv3.0rc.genome.standard.fa.gz |
|  | *Gossypium hirsutum* | https://www.cottongen.org/cottongen_downloads/Gossypium_hirsutum/WHU-TM1_AD1_Updated/assembly/Ghirsutum_TM-1_WHU_genome.standard.fa.gz |
|  | *Gossypium barbadense* | https://www.cottongen.org/cottongen_downloads/Gossypium_barbadense/HEAU-Pima90_AD2genome/assembly/Pima90.fa.gz |
|  | *Gossypium raimondii* | https://figshare.com/ndownloader/files/25304561 |
| *Papaver* | *Papaver rhoeas* | https://ngdc.cncb.ac.cn/gwh/Assembly/17874/show, GWHAZPH00000000 |
|  | *Papaver somniferum* | https://ngdc.cncb.ac.cn/gwh/Assembly/17875/show |
|  | *Papaver setigerum* | https://ngdc.cncb.ac.cn/gwh/Assembly/17873/show |
