# Supplementary material for: Deciphering Complex Interactions Between LTR Retrotransposons and Three Papaver Species Using LTR_Stream
Source: Genomics Proteomics Bioinformatics. 2025 Jul 8;23(4):qzaf061. doi: 10.1093/gpbjnl/qzaf061 (PMC12582370; doi:10.1093/gpbjnl/qzaf061)
Supplement: qzaf061_Supplementary_Data [file qzaf061_supplementary_data.zip › Table S3.docx]

**Table S3 Lineage-level classification of the LTR-RTs of the three *Papaver* species**

| **LTR-RT lineage** | **No. of LTR-RTs in *P. rhoeas*** | **No. of LTR-RTs in *P. somniferum*** | **No. of LTR-RTs in *P. setigerum*** | **Total** |
| --- | --- | --- | --- | --- |
| *Retand* | 5915 | 2076 | 2208 | 10,199 |
| *Ale* | 798 | 1324 | 1999 | 4121 |
| *Athila* | 124 | 2133 | 1657 | 3914 |
| Unknown | 790 | 924 | 866 | 2580 |
| *Ivana* | 337 | 786 | 949 | 2072 |
| *CRM* | 506 | 478 | 610 | 1594 |
| *Reina* | 497 | 469 | 595 | 1561 |
| *TAR* | 63 | 411 | 688 | 1162 |
| *Bianca* | 329 | 214 | 363 | 906 |
| *Ogre* | 353 | 167 | 192 | 712 |
| *Tork* | 203 | 172 | 252 | 627 |
| *Ikeros* | 52 | 86 | 107 | 245 |
| mixture | 133 | 13 | 23 | 169 |
| *SIRE* | 30 | 30 | 33 | 93 |
| *Alesia* | 19 | 11 | 27 | 57 |
| *Tekay* | 34 | 6 | 7 | 47 |
| *Galadriel* | 21 | 7 | 8 | 36 |
| *Angela* | 2 | 0 | 2 | 4 |
| *Gymco-III* | 1 | 0 | 0 | 1 |
| *Osser* | 1 | 0 | 0 | 1 |
| No result | 2417 | 3537 | 2966 | 8920 |
| Total | 12,625 | 12,844 | 13,552 | 39,021 |
