# Supplementary material for: Deciphering Complex Interactions Between LTR Retrotransposons and Three Papaver Species Using LTR_Stream
Source: Genomics Proteomics Bioinformatics. 2025 Jul 8;23(4):qzaf061. doi: 10.1093/gpbjnl/qzaf061 (PMC12582370; doi:10.1093/gpbjnl/qzaf061)
Supplement: qzaf061_Supplementary_Data [file qzaf061_supplementary_data.zip › Table S4.docx]

**Table S4 Lineage-level classification of the LTR-RTs of the four *Gossypium* species**

| **LTR-RT lineage** | **No. of LTR-RTs in *G. barbadense*** | **No. of LTR-RTs in *G. herbaceum*** | **No. of LTR-RTs in *G. hirsutum*** | **No. of LTR-RTs in *G. raimondii*** | **Total** |
| --- | --- | --- | --- | --- | --- |
| *Tekay* | 3208 | 4967 | 4407 | 48 | 12,630 |
| *Tork* | 1854 | 645 | 2098 | 243 | 4840 |
| *CRM* | 1097 | 109 | 1370 | 125 | 2701 |
| *Ivana* | 864 | 163 | 866 | 212 | 2105 |
| *Ale* | 654 | 308 | 636 | 281 | 1879 |
| unknown | 270 | 220 | 524 | 4 | 1018 |
| *Athila* | 313 | 285 | 295 | 54 | 947 |
| *Galadriel* | 286 | 78 | 305 | 127 | 796 |
| *Ogre* | 210 | 106 | 236 | 12 | 564 |
| *Reina* | 88 | 45 | 85 | 43 | 261 |
| *TAR* | 114 | 14 | 104 | 27 | 259 |
| *Bianca* | 87 | 58 | 87 | 14 | 246 |
| *Ikeros* | 36 | 32 | 36 | 3 | 107 |
| *Angela* | 38 | 8 | 33 | 11 | 90 |
| *Alesia* | 12 | 5 | 9 | 5 | 31 |
| mixture | 5 | 1 | 2 | 0 | 8 |
| *SIRE* | 0 | 0 | 1 | 1 | 2 |
| *Selgy* | 0 | 1 | 0 | 0 | 1 |
| No result | 283 | 516 | 331 | 32 | 1162 |
| Total | 9419 | 7561 | 11,425 | 1242 | 29,647 |
