# Supplementary figures and images for: Deciphering Complex Interactions Between LTR Retrotransposons and Three Papaver Species Using LTR_Stream
Source: Genomics Proteomics Bioinformatics. 2025 Jul 8;23(4):qzaf061. doi: 10.1093/gpbjnl/qzaf061 (PMC12582370; doi:10.1093/gpbjnl/qzaf061)

# Self BLAST results

Location1

Location2

e-value

A

B

1E-15

C

D

1E-20

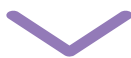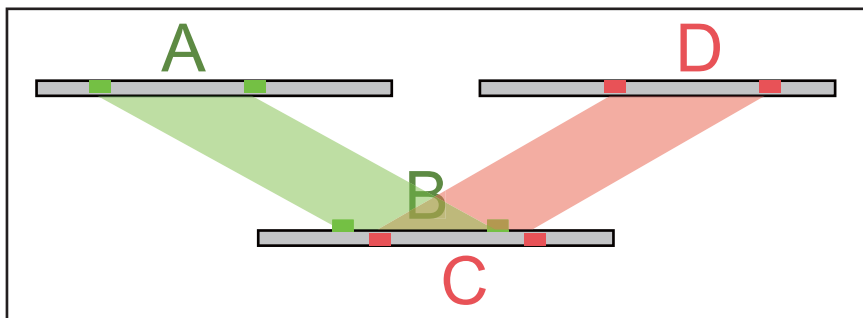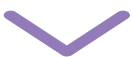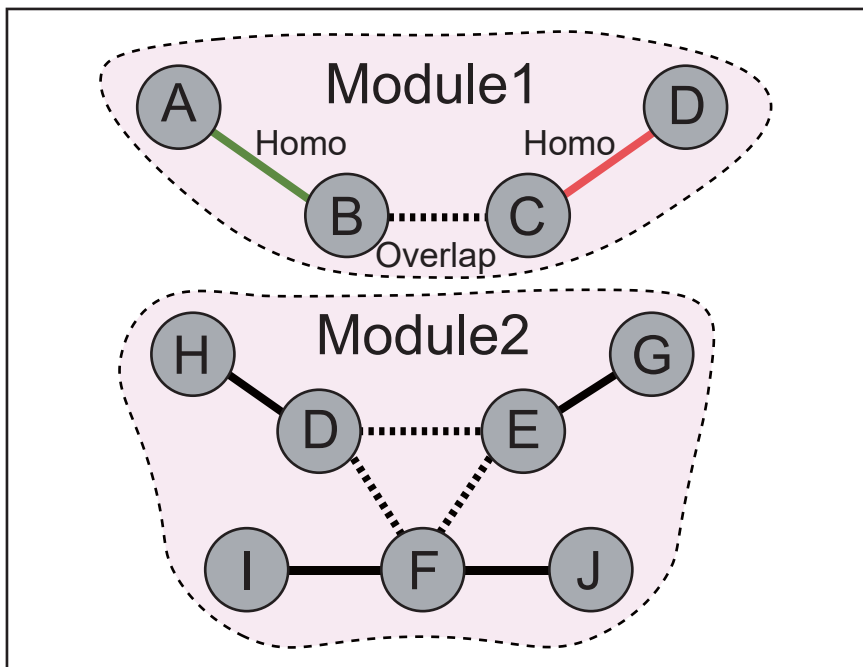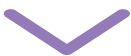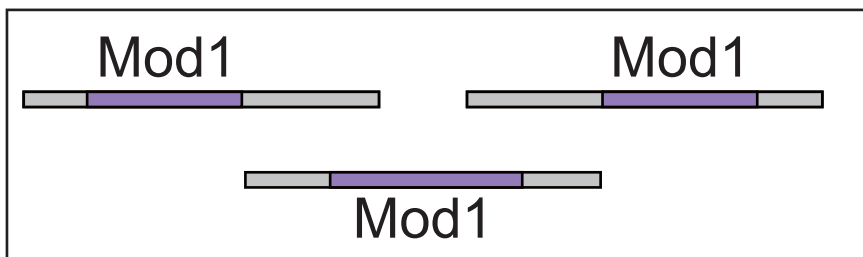

Supplement: qzaf061_Supplementary_Data [file qzaf061_supplementary_data.zip › Fig S1.pdf]

A

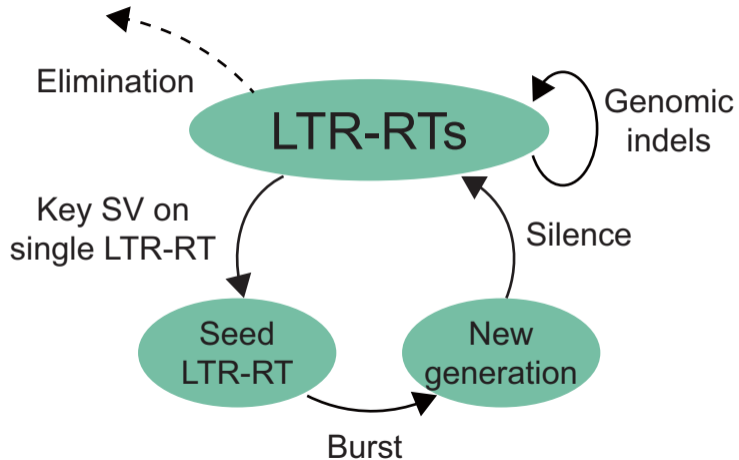

B

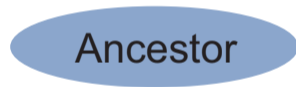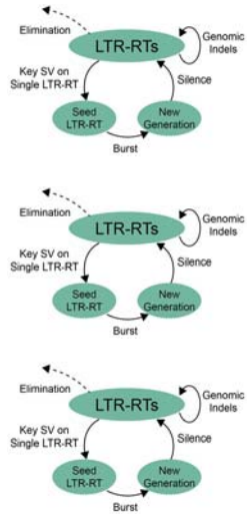

Supplement: qzaf061_Supplementary_Data [file qzaf061_supplementary_data.zip › Fig S2.pdf]

A

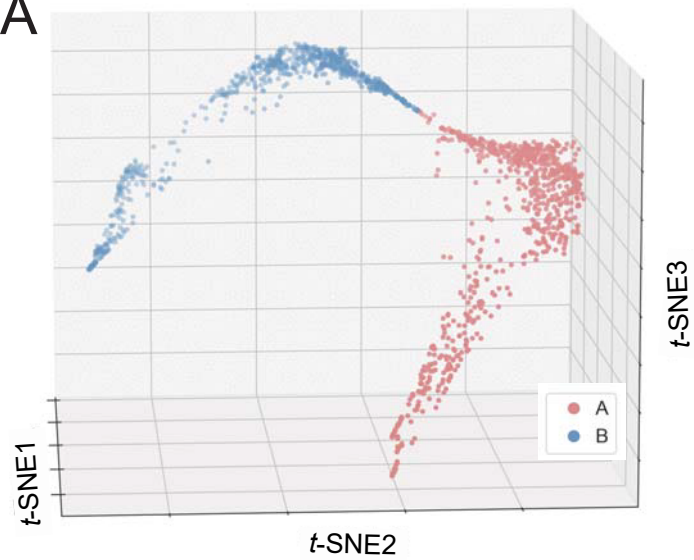

B

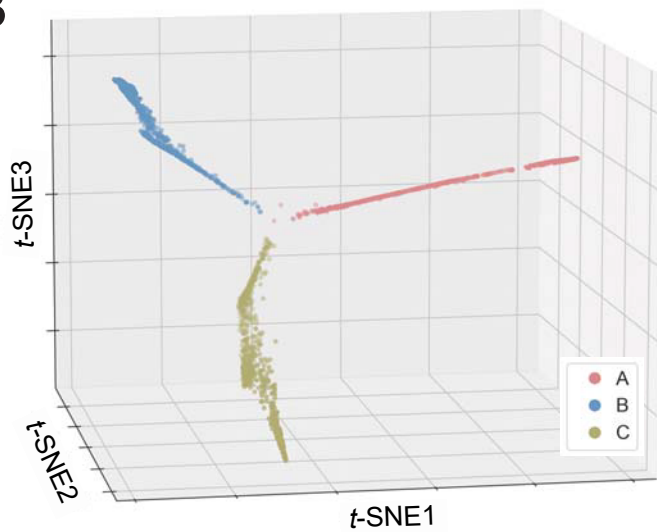

C

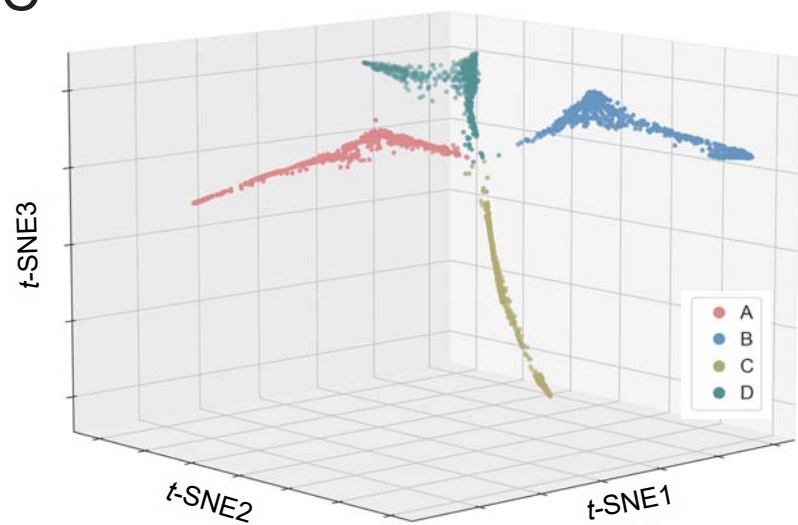

Supplement: qzaf061_Supplementary_Data [file qzaf061_supplementary_data.zip › Fig S3.pdf]

**A**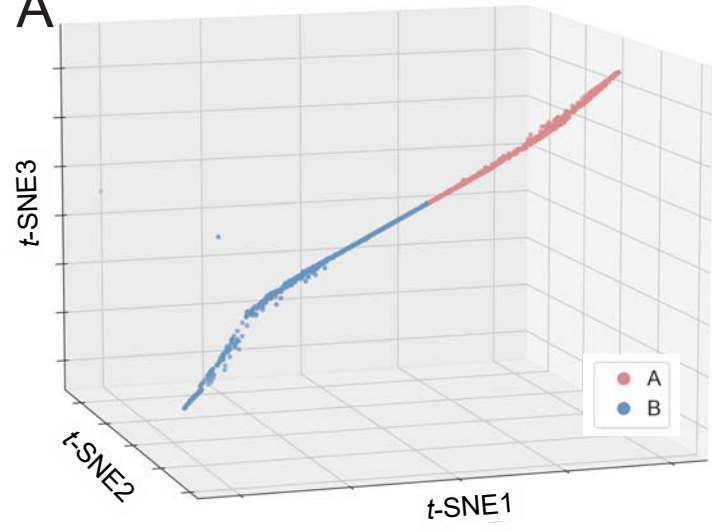**B**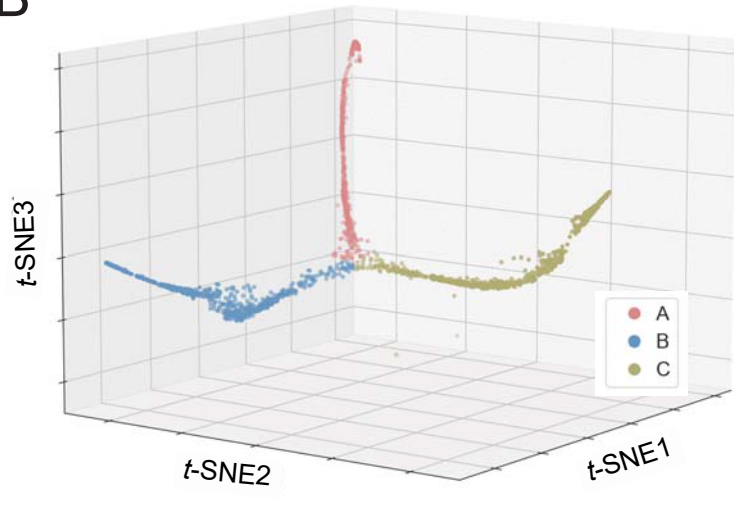

Supplement: qzaf061_Supplementary_Data [file qzaf061_supplementary_data.zip › Fig S4.pdf]

A

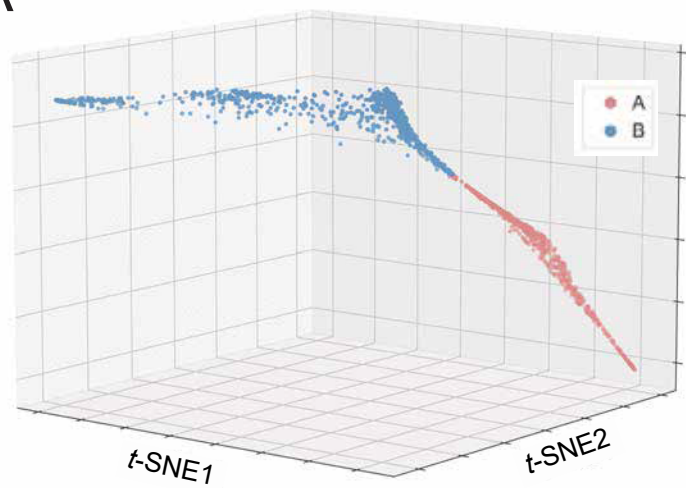

B

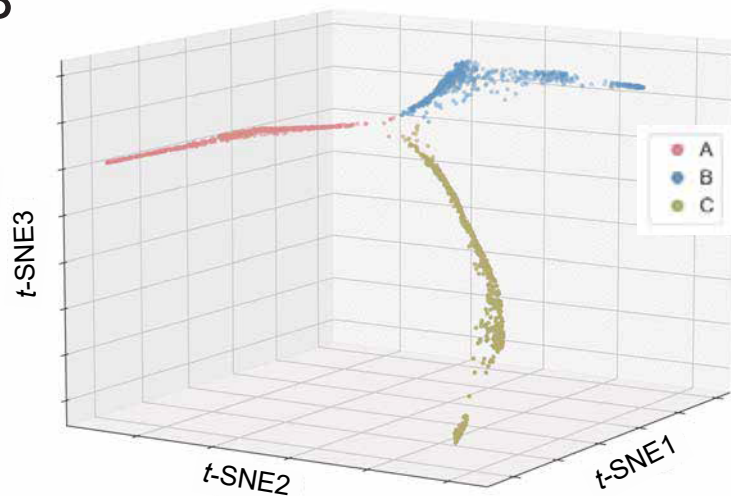

C

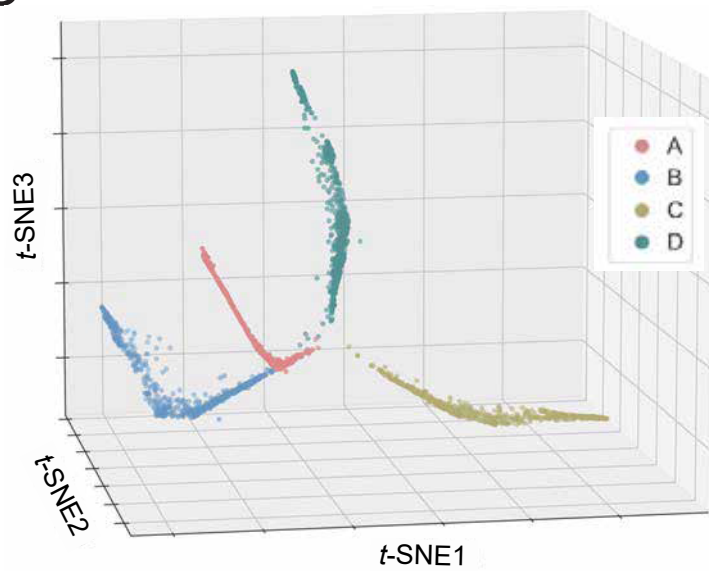

Supplement: qzaf061_Supplementary_Data [file qzaf061_supplementary_data.zip › Fig S5.pdf]

A

*Papaver Ale*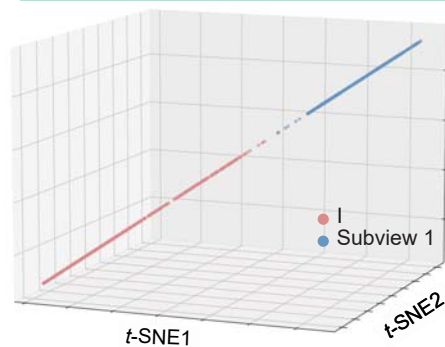

+

Subview 1

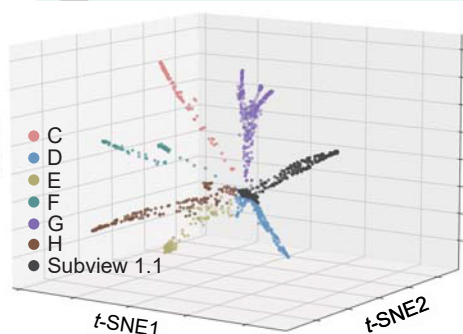

+

Subview 1.1

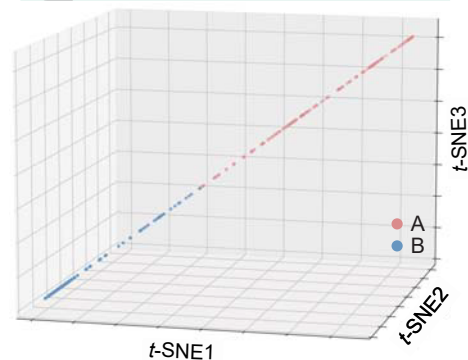

B

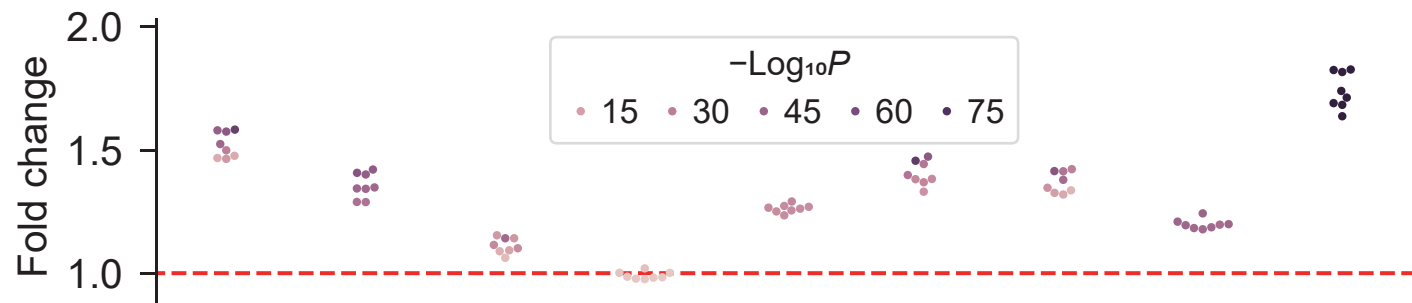

C

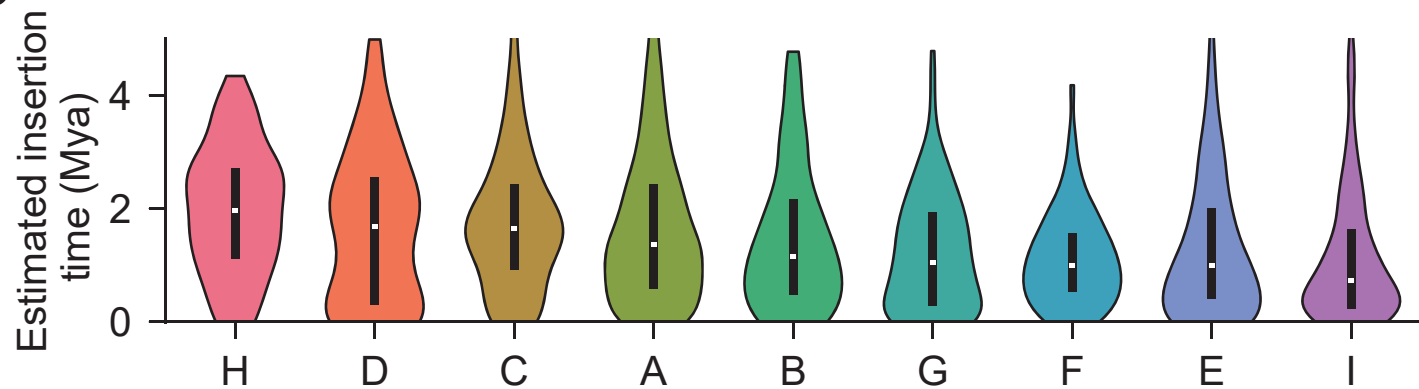

Supplement: qzaf061_Supplementary_Data [file qzaf061_supplementary_data.zip › Fig S6.pdf]

A

## Cotton Tekay

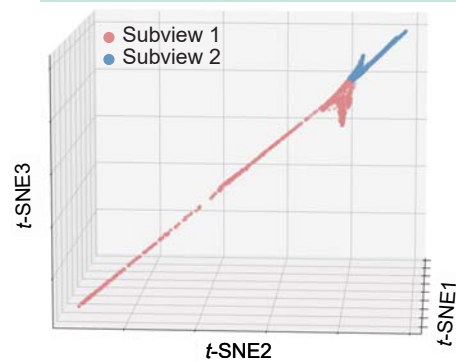

+

## Subview 1

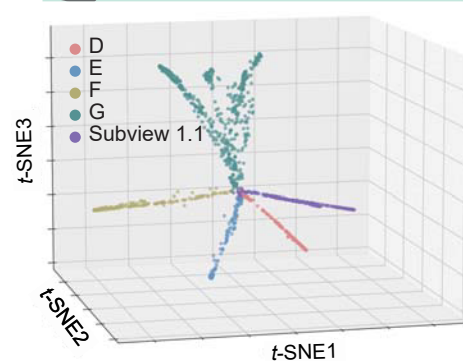

+

## Subview 1.1

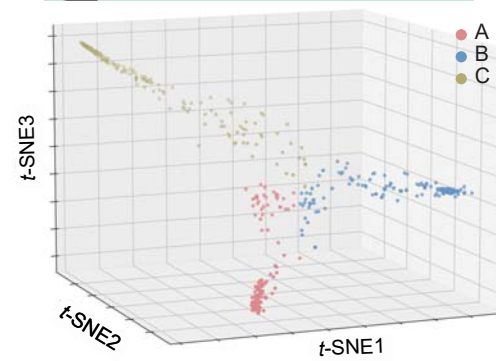

+

## Subview 2

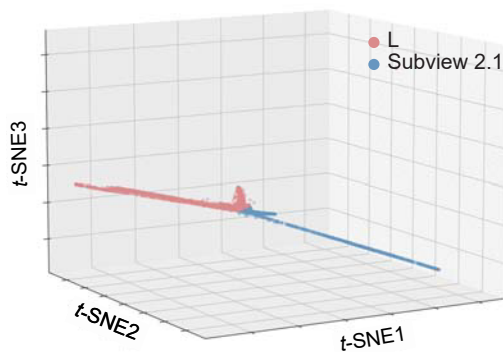

+

## Subview 2.1

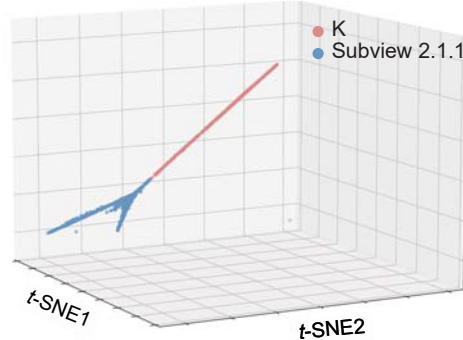

+

## Subview 2.1.1

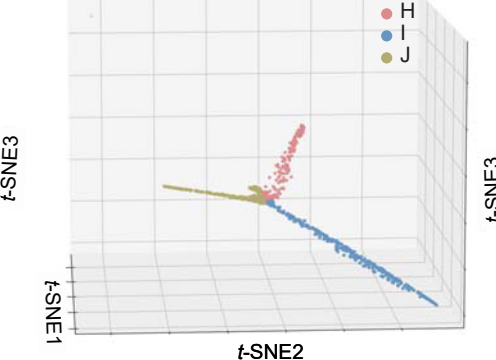

B

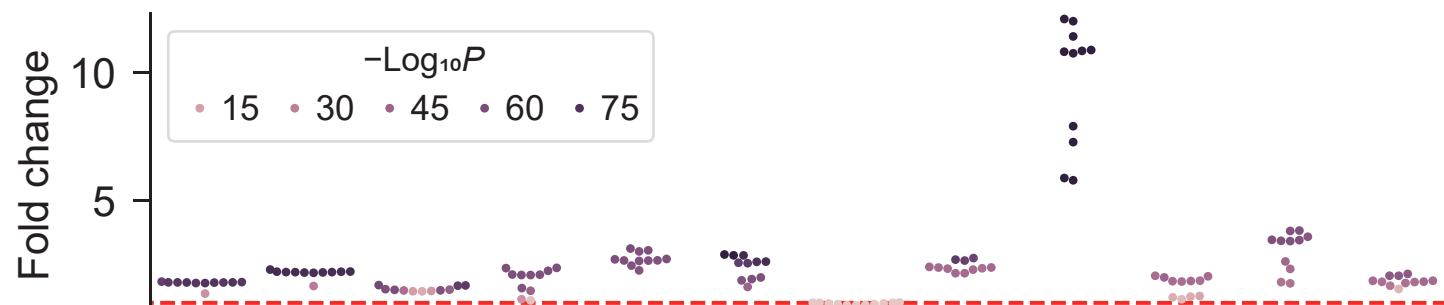

C

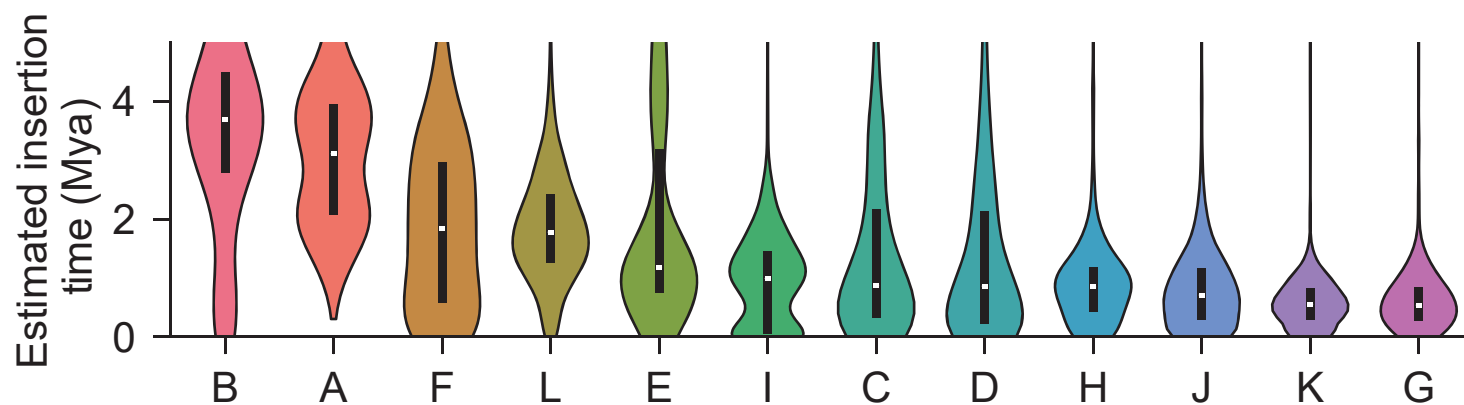

Supplement: qzaf061_Supplementary_Data [file qzaf061_supplementary_data.zip › Fig S7.pdf]

A

*Papaver CRM*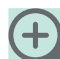

Subview 1

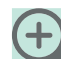

Subview 2

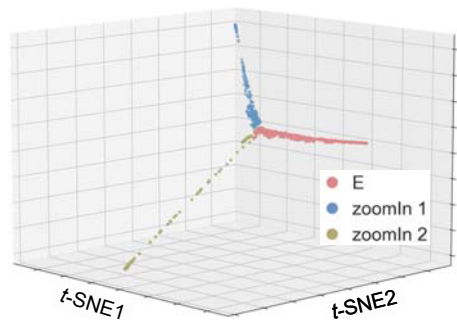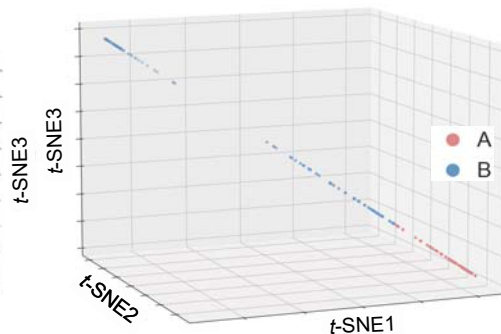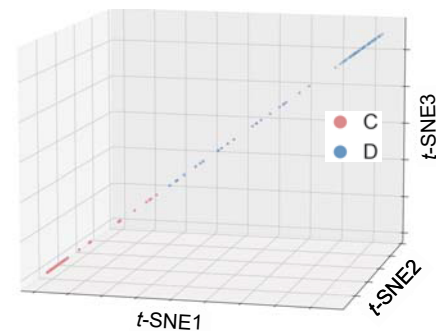

B

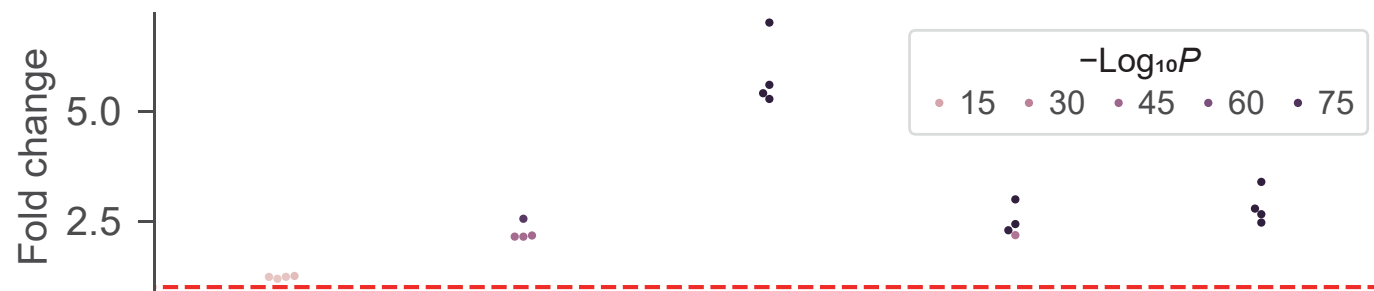

C

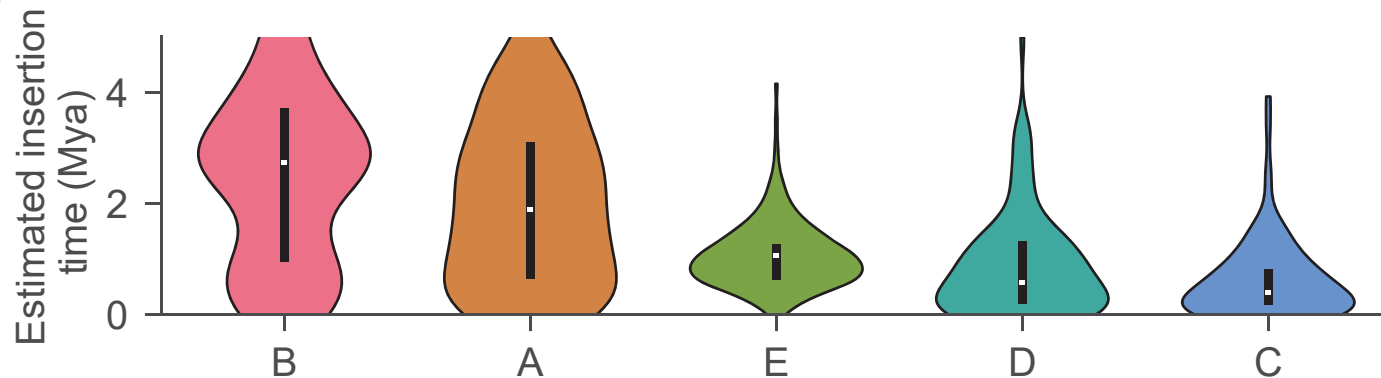

Supplement: qzaf061_Supplementary_Data [file qzaf061_supplementary_data.zip › Fig S8.pdf]

A

*Papaver Ogre*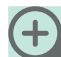

Subview 1

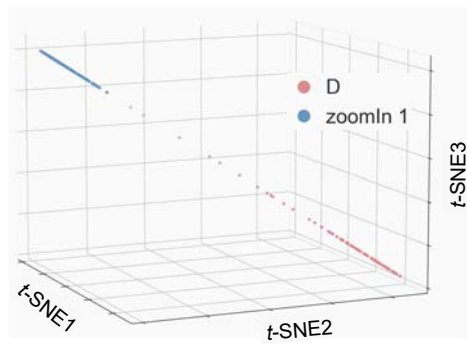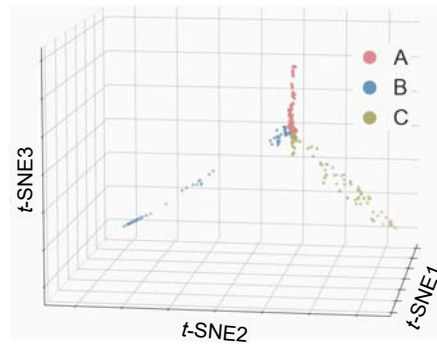

B

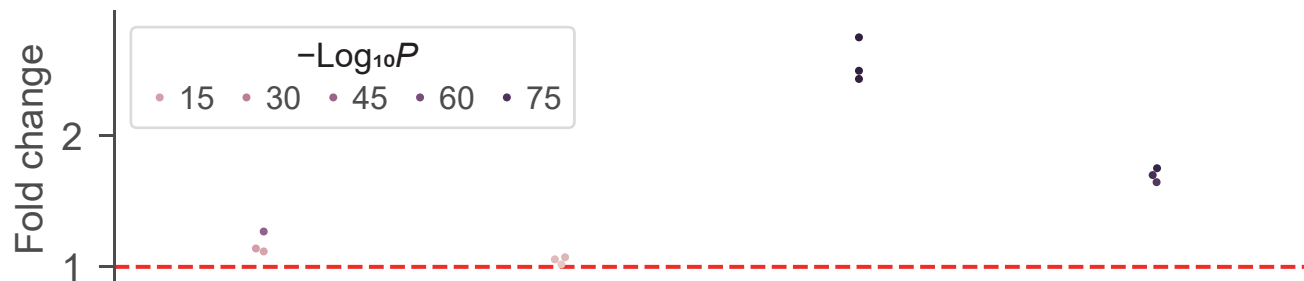

C

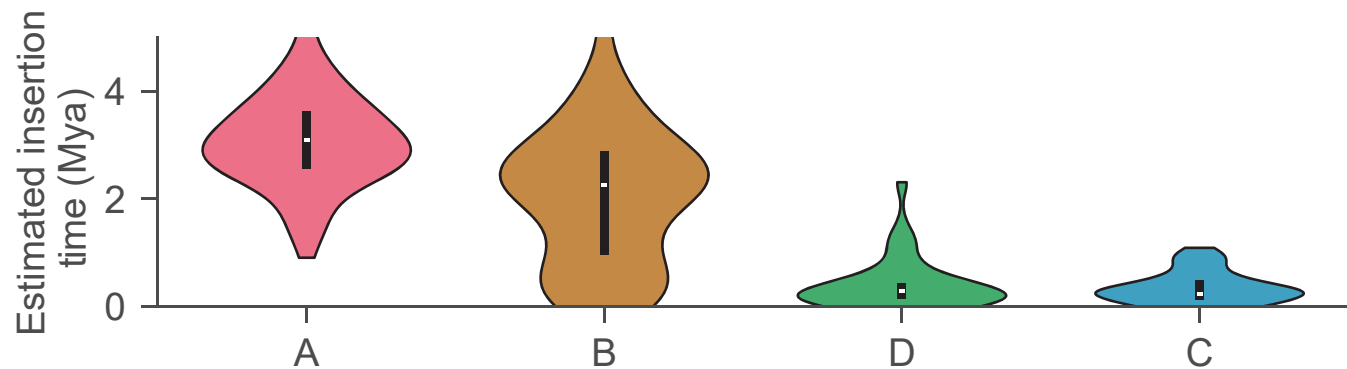

Supplement: qzaf061_Supplementary_Data [file qzaf061_supplementary_data.zip › Fig S9.pdf]

A

# *Papaver Tork*

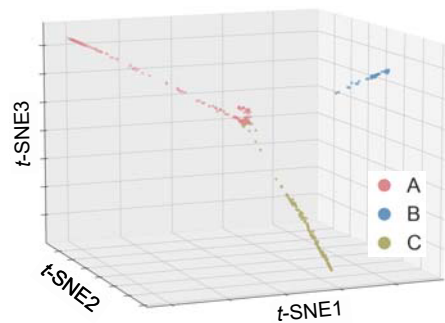

B

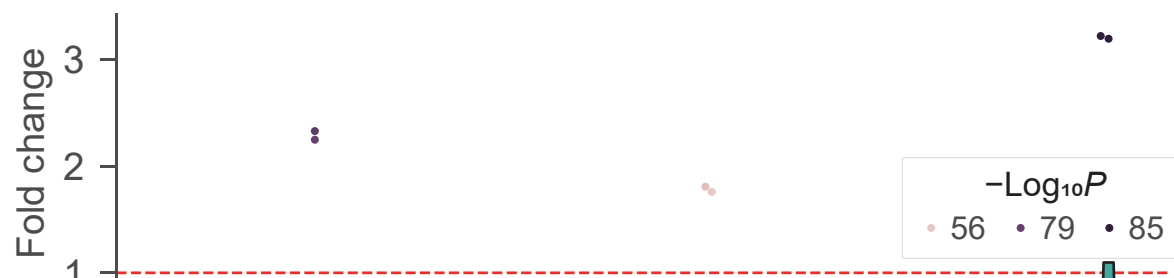

C

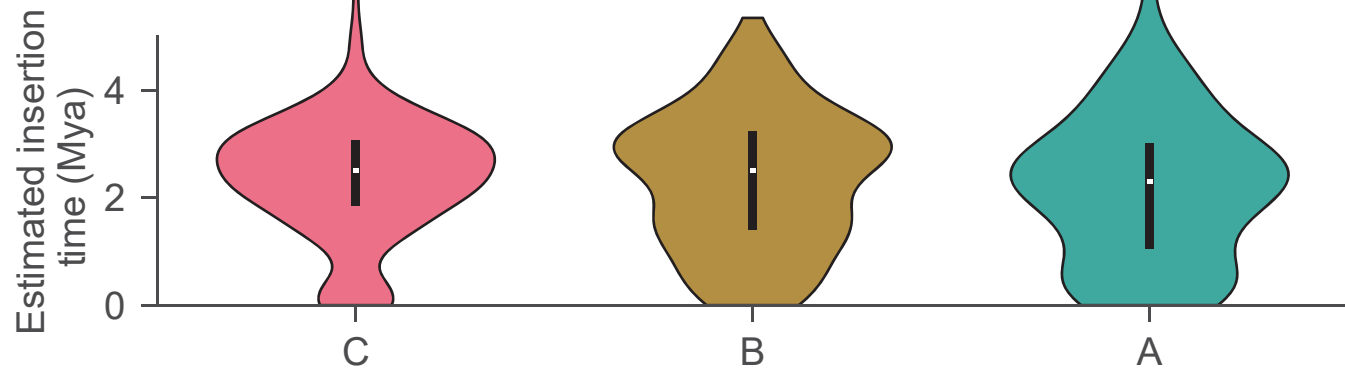

Supplement: qzaf061_Supplementary_Data [file qzaf061_supplementary_data.zip › Fig S10.pdf]

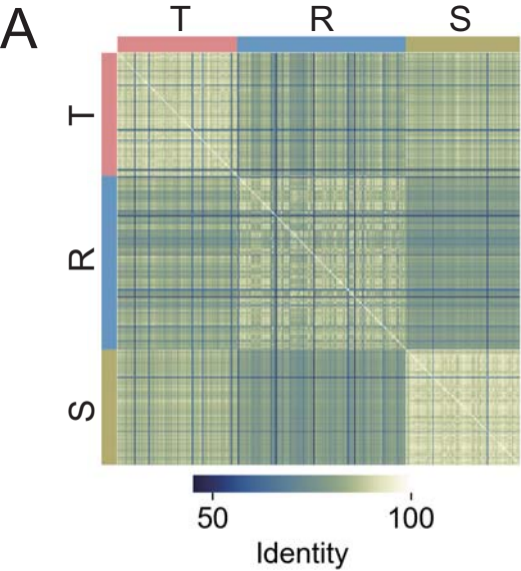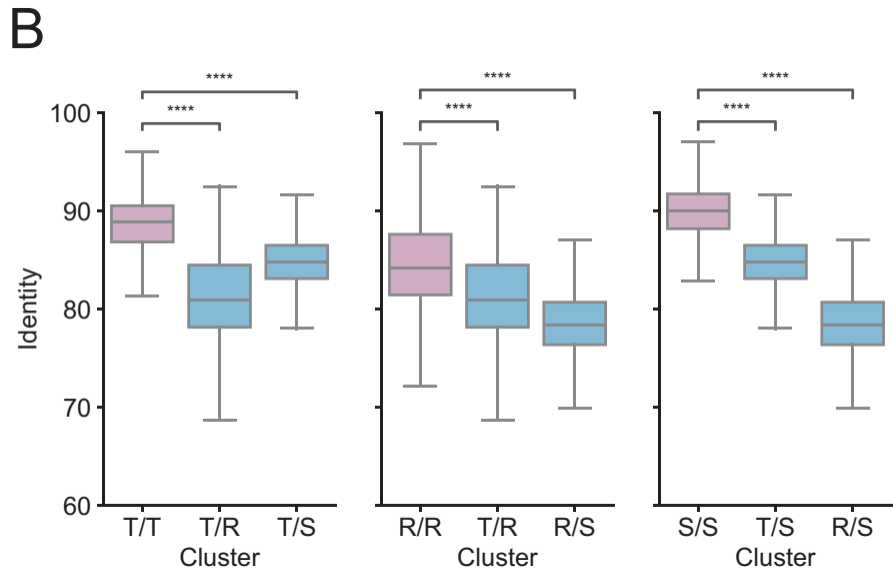

Supplement: qzaf061_Supplementary_Data [file qzaf061_supplementary_data.zip › Fig S12.pdf]

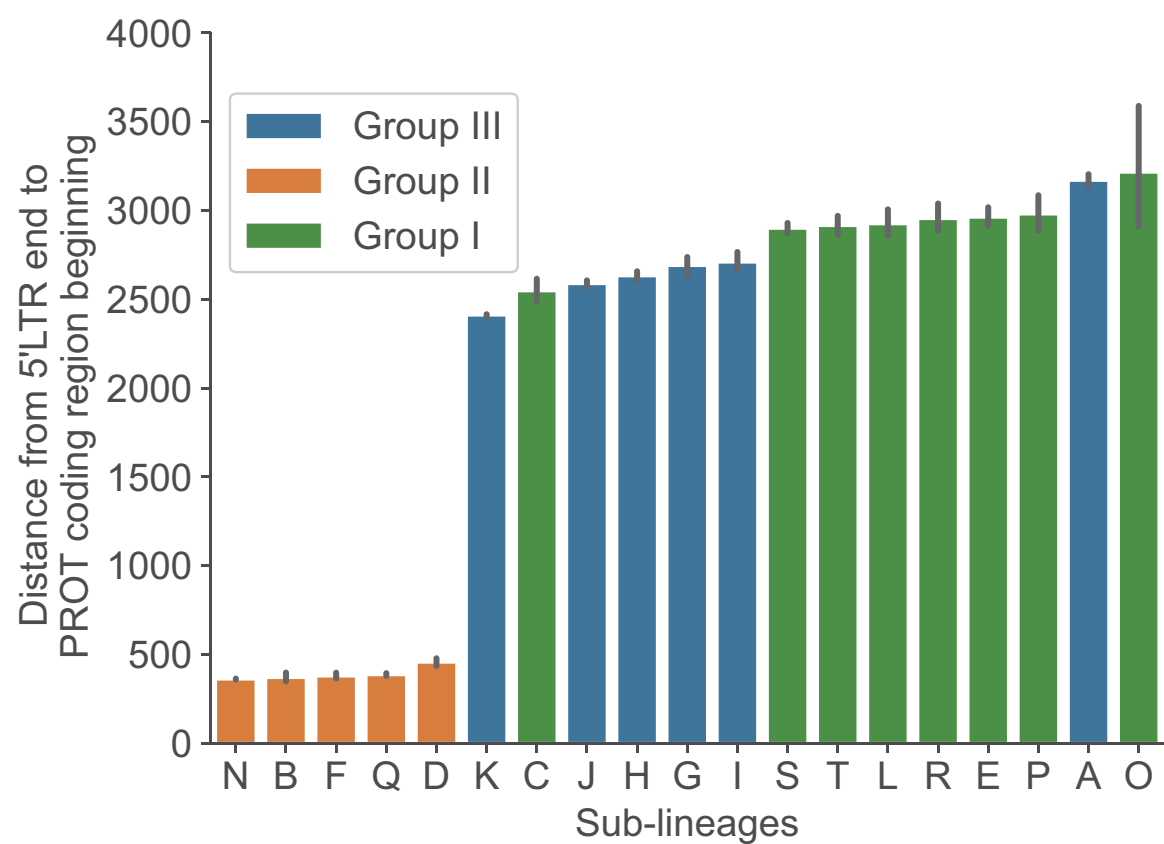

Supplement: qzaf061_Supplementary_Data [file qzaf061_supplementary_data.zip › Fig S14.pdf]

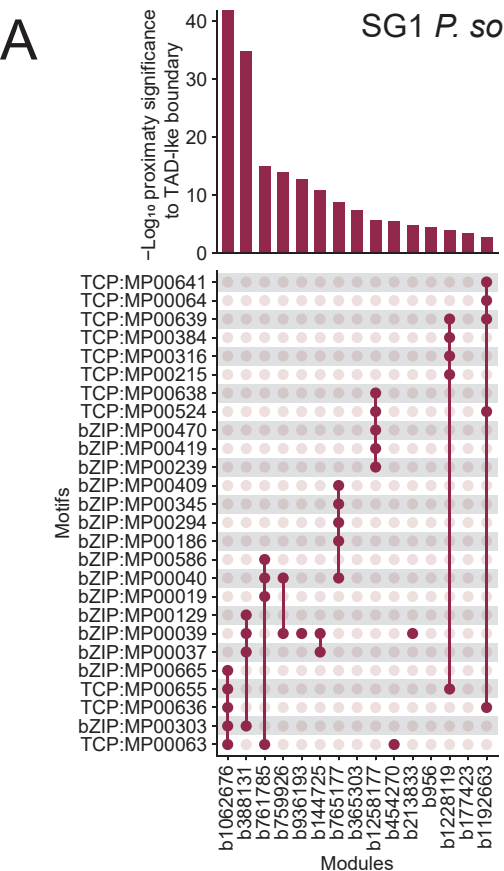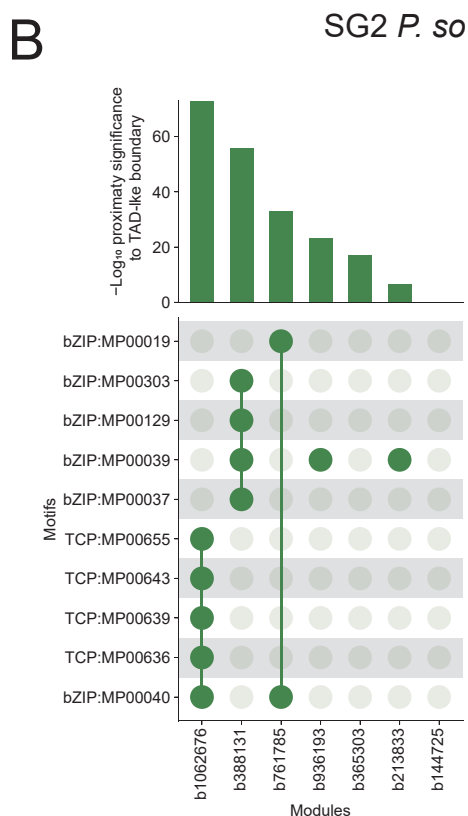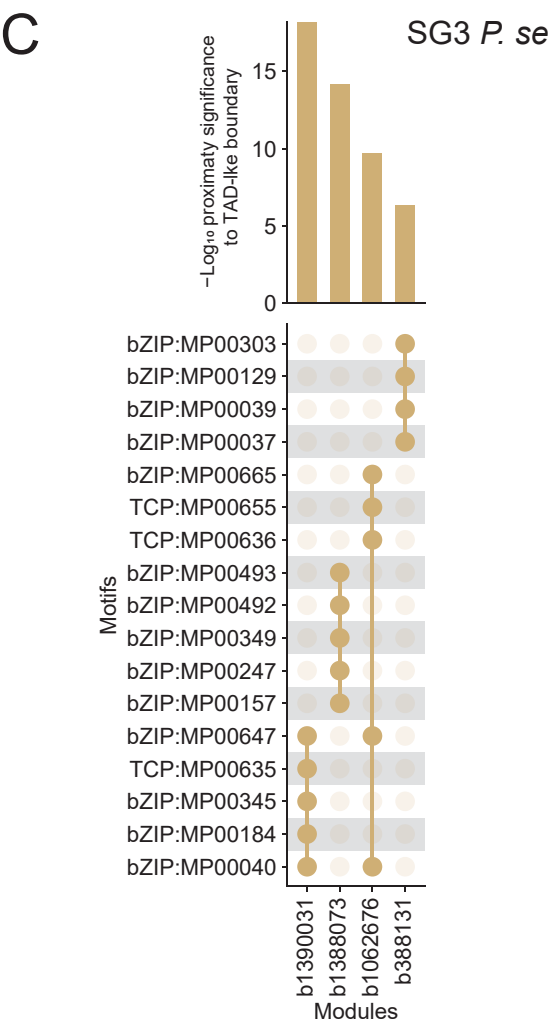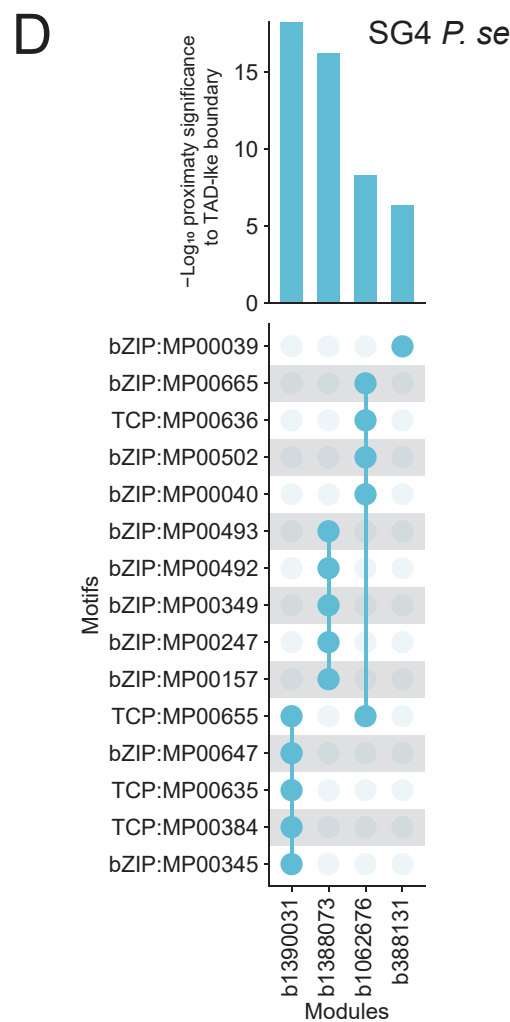

Supplement: qzaf061_Supplementary_Data [file qzaf061_supplementary_data.zip › Fig S15.pdf]

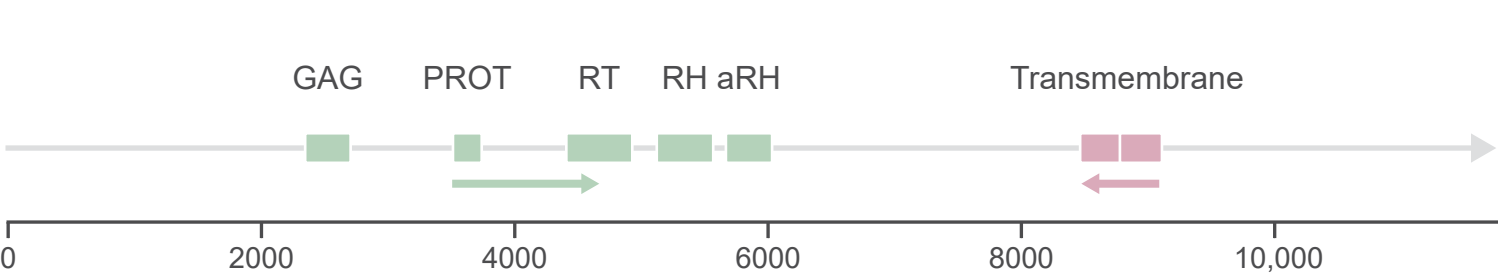

Supplement: qzaf061_Supplementary_Data [file qzaf061_supplementary_data.zip › Fig S16.pdf]
