# Supplementary material for: Deciphering Complex Interactions Between LTR Retrotransposons and Three Papaver Species Using LTR_Stream
Source: Genomics Proteomics Bioinformatics. 2025 Jul 8;23(4):qzaf061. doi: 10.1093/gpbjnl/qzaf061 (PMC12582370; doi:10.1093/gpbjnl/qzaf061)
Supplement: qzaf061_Supplementary_Data [file Fig S11.pdf › Fig S11.pdf]

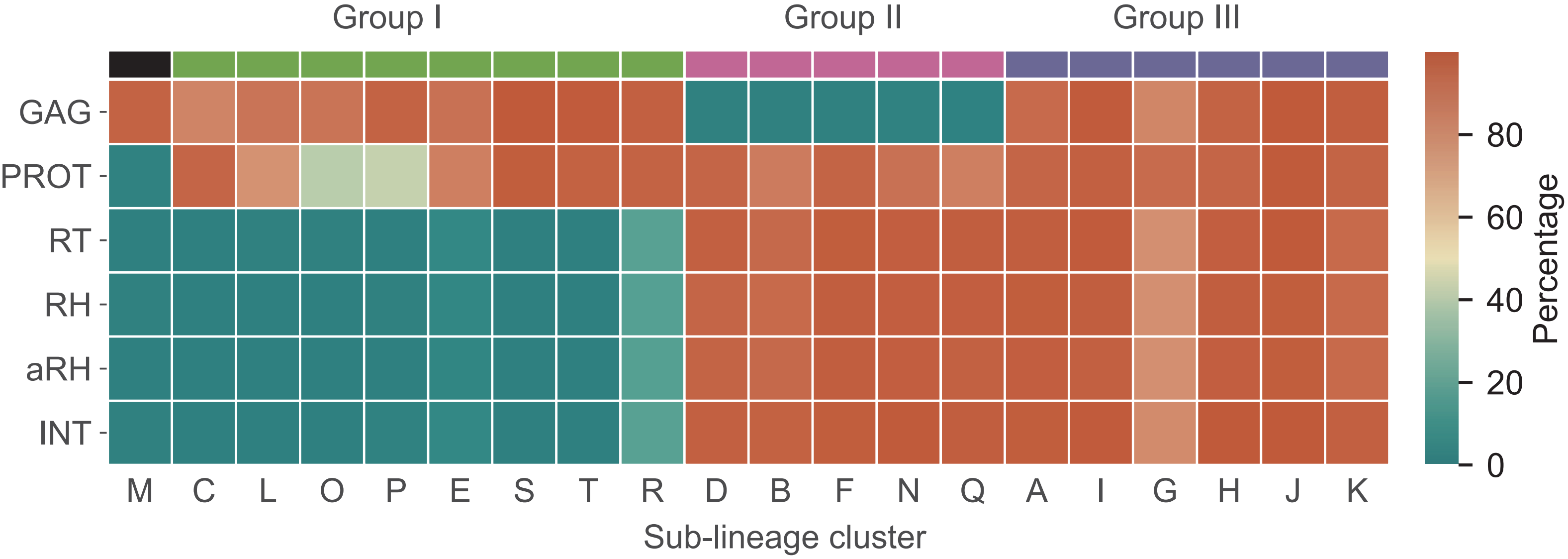

Group I
Group II
Group III
GAG
PROT
RT
RH
aRH
INT
M
C
L
O
P
E
S
T
R
D
B
F
N
Q
A
I
G
H
J
K
Sub-lineage cluster
Percentage
0
20
40
60
80
